# Novel prognostic genes and subclasses of acute myeloid leukemia revealed by survival analysis of gene expression data

**DOI:** 10.1186/s12920-021-00888-0

**Published:** 2021-02-03

**Authors:** Yanli Lai, Guifang OuYang, Lixia Sheng, Yanli Zhang, Binbin Lai, Miao Zhou

**Affiliations:** Department of Hematology, Ningbo First Hospital, 59 Liuting RoadZhejiang Province, Ningbo, 315000 China

**Keywords:** The cancer genome atlas database, Acute myeloid leukemia, Weighted gene co-expression network analysis, Risk score, Overall survival

## Abstract

**Background:**

Acute myeloid leukemia (AML) is biologically heterogeneous diseases with adverse prognosis. This study was conducted to find prognostic biomarkers that could effectively classify AML patients and provide guidance for treatment decision making.

**Methods:**

Weighted gene co-expression network analysis was applied to detect co-expression modules and analyze their relationship with clinicopathologic characteristics using RNA sequencing data from The Cancer Genome Atlas database. The associations of gene expression with patients’ mortality were investigated by a variety of statistical methods and validated in an independent dataset of 405 AML patients. A risk score formula was created based on a linear combination of five gene expression levels.

**Results:**

The weighted gene co-expression network analysis detected 63 co-expression modules. The pink and darkred modules were negatively significantly correlated with overall survival of AML patients. High expression of *FNDC3B, VSTM1* and *CALR* was associated with favourable overall survival, while high expression of *PLA2G4A* was associated with adverse overall survival. Hierarchical clustering analysis of *FNDC3B, VSTM1, PLA2G4A*, *GOLGA3* and *CALR* uncovered four subgroups of AML patients. The cluster1 AML patients showed younger age, lower cytogenetics risk, higher frequency of *NPM1* mutations and more favourable overall survival than cluster3 patients. The risk score was demonstrated to be an indicator of adverse prognosis in AML patients

**Conclusions:**

The *FNDC3B, VSTM1, PLA2G4A*, *GOLGA3, CALR* and risk score may serve as key prognostic biomarkers for the stratification and ultimately guide rational treatment of AML patients.

## Background

Acute myeloid leukemia (AML) is biologically heterogeneous diseases with a relatively adverse survival rate [1]. The Surveillance, Epidemiology, and End Results Program [2], reports an incidence rate of 4.3 per 100,000 persons and mortality rate of 2.8 per 100,000 persons annually. AML patients show a relatively poor 5-year survival rate of 27.4%. The 2017 European Leukemia Net (ELN) guidelines are well established tools for the assessment of risk of resistance and prognosis for AML patients. The ELN 2017 could effectively classify AML patients into three subgroups, including favorable, intermediate and poor subgroups, according to leukemia cell genetic abnormalities and mutations in driver genes [3]. For instance, some cytogenetic abnormalities are related to favorable clinical outcome, such as inv(16)(p13.1q22) and t(8;21)(q22;q22.1). While, others are indicative of poor overall survival in AML patients, such as t(6;9)(p23;q34.1), inv(3)(q21.3q26.2) [3]. *RUNX1-RUNX1T1* or *MYH11-CBFB* fusions are indicators of good clinical outcomes in AML patients who underwent chemotherapy based consolidation regimens [4, 5]. While, a large proportion of AML genomes are lack of structural abnormalities [6, 7]. In addition to cytogenetic abnormalities, the 2017 ELN also includes mutations in several genes for risk stratification. The *TP53* mutation is one of the known adverse factors and frequently associated with complex cytogenetics. *NPM1* and *CEBPA* mutations are indicative of favorable prognosis regardless of cytogenetic abnormalities. A FLT3 internal tandem duplication (ITD) with the ratio of mutated to normal alleles > 0.5 is associated with poor prognosis [3]. *DNMT3A*, *NPM1* mutations and *MLL* translocations have been shown to ameliorate risk classification for patients showing normal karyotype [8]. However, these genes are not applied to those AML patients who didn’t have *DNMT3A*, *NPM1* mutations and *MLL* translocations [8]. Therefore, none of the current markers is entirely accurate, novel biomarkers are required to improve prognostic classification.

The weighted gene co-expression network analysis (WGCNA) package identifies co-expression modules in which the expression of a set of genes is highly correlated and seeks for associations between interested co-expression modules and clinical characteristics. The analysis enables researchers to detect co-expression networks related to certain phenotypic trait [9]. In this study, we applied the WGCNA algorithm to a genome-wide study of 18,366 genes using RNA-seq expression data of 173 AML patients from The Cancer Genome Atlas (TCGA) database. The WGCNA analysis revealed two co-expression modules which were significantly associated with patients’ overall survival (OS). Further analysis of the two mortality-associated modules identified a gene panel of *FNDC3B, VSTM1, PLA2G4A*, *GOLGA3* and *CALR.* Hierarchical clustering analysis of the five genes enabled the identification of a subgroup of AML patients with favourable OS. The co-expression modules and gene panel may be of importance in evaluating the prognosis of AML patients.

## Methods

### Data acquisition and processing

In total, normalized read counts (RNA-seq) data of 20,531 genes of 173 AML patients and their clinical data were acquired from the TCGA database [10]. Genes without expression values in 90% AML patients were removed. Totally, 18,366 genes met the inclusion criterion of the WGCNA analysis. The FAB subtypes consisted of 8 subtypes, including minimal maturation AML (M0), no maturation AML (M1), maturation AML (M2), acute promyelocytic (M3), myelomonocytic (M4), monoblastic or monocytic (M5), erythroid (M6), megakaryoblastic (M7) leukemia and others. Cytogenetic risk comprised favorable, intermediate and poor prognosis categories. Gene expression and clinical characteristics of AML patients (n = 405) were obtained from the Oregon Health & Science University (OHSU) database for validation analysis [11].

### The weighted gene co-expression network analysis in AML

Co-expression networks were built by the R package of WGCNA using normalized read count data of 18,366 genes of 173 AML patients in R3.2.0. The parameter of soft thresholding was set to 7, the minimum number of genes was set to 30, other parameters were used with the default values. Heatmap tools package was painted to analyze the strength of the interactions. The constructed modules were ranged by the number of genes and genetic information was extracted from each module. In order to identify co-expression modules which showed significant correlation with phenotypes, associations between modules and clinical traits were investigated by analyzing the correlation of the co-expression module eigengenes with clinical traits.

### Functional enrichment analysis

We utilized the Gene ontology (GO) [12] and Search Tool for the Retrieval of Interacting Genes/Proteins (STRING)[13] to analyze the potential functional importance for the genes in the co-expressed modules. The enrichment of GO terms and Kyoto Encyclopedia of Genes and Genomes (KEGG) pathways were regarded to be statistically significant based on the cutoff values of adjusted *P* value and false discovery rate (FDR) < 0.05 respectively.

### Survival analyses

We followed the methods of Lai et al. and Sha et al. to perform the survival analyses [14, 15]. In brief, AML patients were grouped into two subgroups, including high and low expression groups, according to the cutoff values determined by the pROC package [16]. The difference in overall survival rates was compared between the two groups of AML patients using the Kaplan–Meier survival analysis. The prognostic importance of genes were further evaluated by the logistic regression model [17, 18]. Survival-related genes were further divided into risk genes (odd ration [OR] > 1) and protective genes (0 < OR < 1). The five genes *FNDC3B, VSTM1, PLA2G4A*, *GOLGA3* and *CALR* were used to build the risk score model. Risk score = expression of gene 1 × β1 + expression of gene 2 × β2 + ⋯ + expression of gene n × βn. The β values were coefficients generated by the logistic regression model. The relation between risk scores and OS was investigated by Kaplan–Meier survival analysis and logistic regression model analysis. *P* < 0.05 was considered statistically significant.

### Unsupervised hierarchical clustering analysis

Unsupervised hierarchical clustering of *CALR, VSTM1*, *PLA2G4A, GOLGA3* and *FNDC3B* was conducted with the R package of pheatmap [19]. Difference in quantitative clinical factors was analyzed by analysis of variance test among four clusters of patients. For between-group comparison, the Wilcoxon sum rank test was used. Difference in qualitative variables was investigated by fisher exact test. To characterize the prognostic probabilities of clusters of AML patients, we plotted Kaplan–Meier curves and compared overall survival rate differences using the log-rank test [20]. *P* < 0.05 was considered statistically significant.

## Results

### General characteristics of 173 AML patients

The mean age was 55.28 (range 18–88 years old). The mean percent of bone marrow blast cells was 38.85 at diagnosis. The AML patients comprised 16 M0, 42 M1, 39 M2, 16 M3, 35 M4, 18 M5, 2 M6, 3 M7 and 2 unclassified samples. 32, 103 and 36 patients were predicted to have favorable, intermediate and poor prognosis by the ELN guidelines respectively. The number of AML patients with *IDH1, IDH2, DNMT3A, NPM1, FLT3* and *CEBPA* mutations was 16, 17, 43, 48, 48 and 13 respectively. 45 AML patients received neoadjuvant treatment. 114 AML patients were dead, 59 were alive and 10 patients were lost to contact. The average follow-up time was 563.61 days (range 0–2861 days).

### Detection of co-expression modules in AML

**Fig. 1:**
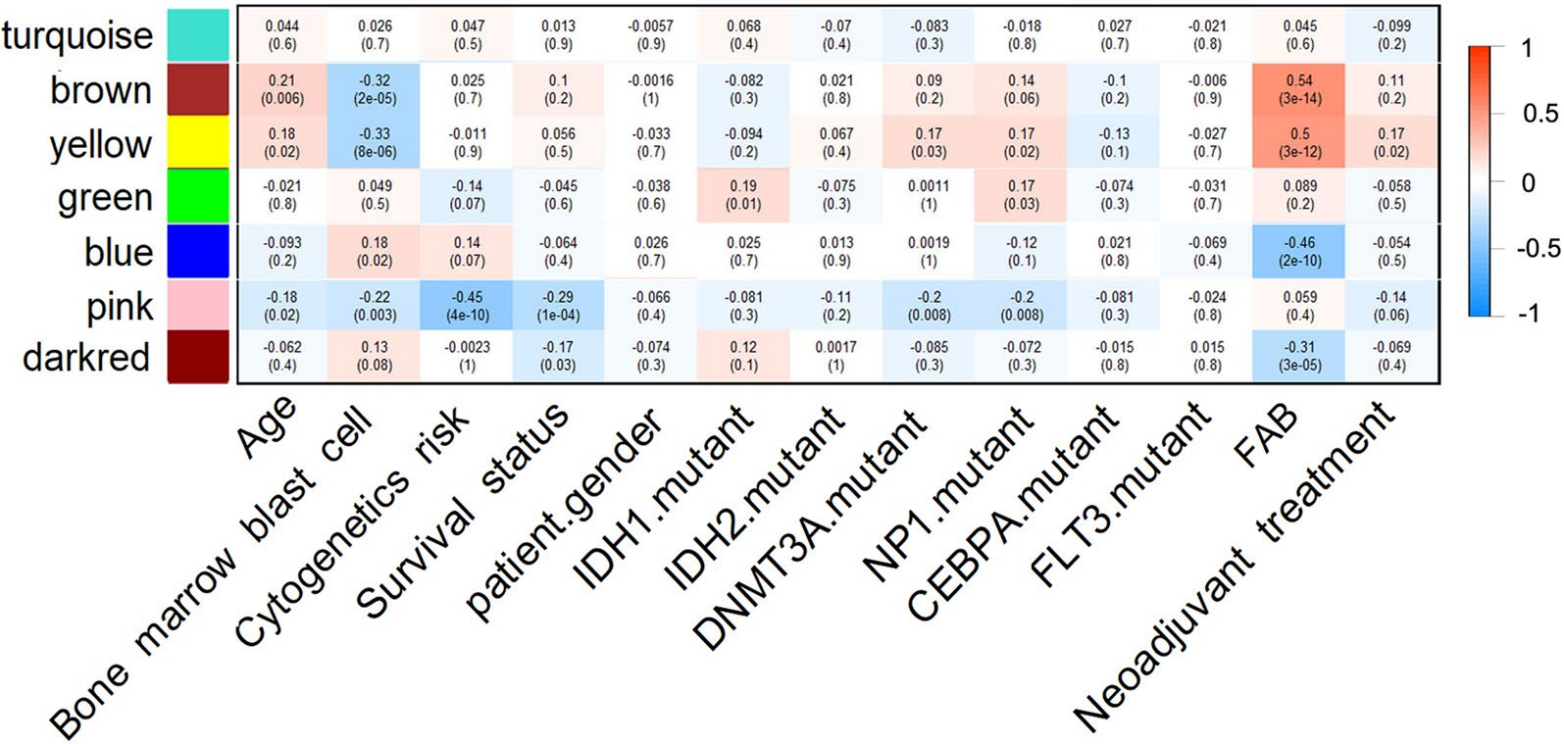
The associations between co-expression modules and clinical traits. Each row and column corresponded to a module eigengene and clinical trait respectively. The correlation co-efficient and *P* value were presented in each cell. The red-to-blue bar on the right showed the degree of correlation between co-expression modules and clinical traits

The WGCNA package was applied to construct the co-expression network and detect co-expression modules using normalized read counts of 18,366 genes of the 173 AML samples. The scale-free fit index was greater than 0.8 and the mean connectivity of the WGCNA network was stable at the soft-thresholding power value of six. Therefore, we used the soft-thresholding power value of seven in the WGCNA analysis (Additional file 1: Figure 1). The WGCNA analysis detected 63 co-expression modules and turquoise, blue, brown, yellow and green modules were the top 5 modules having largest number of genes (Fig. 1 and Additional file 1: Figure 2, Additional file 2: Table 1).

### Module-trait association analysis in AML

The majority of modules (59/63) showed significant correlation with the 15 clinical traits. 14, 26, 22, 5, 3, 3, 6,23, 2,12,27 and 12 modules were significantly correlated with patients’ age, bone marrow blast cell, cytogenetic risk, gender, *IDH1* mutation, *IDH2* mutation, *DNMT3A* mutation, *NPM1* mutation, *CEBPA* mutation, *FLT3* mutation, FAB subtypes and neoadjuvant treatment respectively (*P* value < 0.05 for all cases, Fig. 1 and Additional file 2: Table 1). Importantly, the pink and darkred modules (hereinafter referred to as overall survival-associated module1: OSAM1, overall survival-associated module2: OSAM2 respectively) were negatively correlated with patients’ OS (*P* value < 0.05 for all cases, Fig. 1). Moreover, the OSAM1 module also showed significantly negative correlation with patients’ age, PBMBC, cytogenetic risk, *DNMT3A* mutation and *NPM1* mutation. The OSAM2 module was negatively correlated with FAB subtypes (*P* value < 0.05 for all cases, Fig. 1, Table1).

**Table 1:** The associations between clinical traits and the OSAM1 and OSAM2 in the WGCNA network

Module	Number of genes	Correlation with clinical traits
OSAM1	429	Age, PBMBC, Cytogenetic risk, OS, DNMT3A mutation, NP1 mutation
OSAM2	151	OS, FAB

*PBMBC* percent of bone marrow blast cells, *OSAM1* overall survival-associated module1, *OSAM2* overall survival-associated module2

### Functional annotation of genes in the OSAM1 and OSAM2 modules

The functional values of genes in the OSAM1 and OSAM2 modules were analyzed by GO and KEGG pathway enrichment analysis. Genes in the OSAM1 module were significantly enriched in 53 GO terms (adjusted *P* value < 0.05), such as negative regulation of signaling (GO:0023057), regulation of cell communication (GO:0010646), negative regulation of developmental process (GO:0051093), cell differentiation (GO:0030154), negative regulation of developmental process (GO:0051093). Moreover, the genes in the OSAM1 module were over-represented in the KEGG pathway of protein processing in endoplasmic reticulum (FDR < 0.05). The OSAM2 module genes were significantly enriched in the KEGG pathway of other types of O-glycan biosynthesis (FDR = 0.001).

### Identification of survival-related genes in AML

**Fig. 2:**
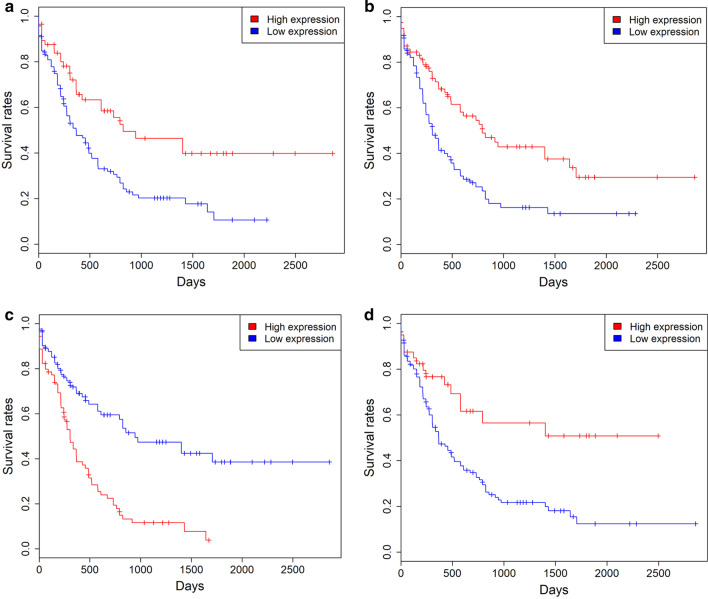
Kaplan–Meier survival analysis of patients’ OS with *VSTM1* (**a**), *FNDC3B* (**b**), *PLA2G4A* (**c**) and *CALR* (**d**) expression levels in 173 AML patients of the TCGA dataset. The blue and red plots are low and high expression groups respectively

Kaplan–Meier survival analysis suggested that patients with high expression levels of 327 genes exhibited favorable clinical outcome, such as *FNDC3B, VSTM1* and *CALR*. Whereas, patients with high expression levels of 12 genes were associated with a poor prognosis, such as *PLA2G4A* (*P* < 0.05 for all cases, log rank test, Fig. 2 and Additional file 2: Table 2). Among the clinicopathologic characteristics, cytogenetic risk and patients’ age were significantly associated with patients’ mortality (*P* < 0.001 for all cases, Fisher exact test or Wilcoxon sum rank test, Additional file 2: Table 3). However, the association was not observed between OS and other factors, such as gender, PBMBC, *IDH1, IDH2, DNMT3A, NPM1, FLT3, CEBPA* mutations and neoadjuvant treatment (*P* > 0.05 for all cases, Fisher exact test or Wilcoxon sum rank test, Additional file 2: Table 3). Then, logistic regression model was applied between patients’ OS and patients’ age, cytogenetic risk, 339 gene expression levels. High expression of 207 genes was associated with favorable prognosis, such as *FNDC3B, VSTM1* and *CALR* (*P* < 0.05 for all cases, OR: 0.32–0.44, Additional file 2: Table 2). While high expression of 8 genes was associated with inferior overall survival, including *IL15RA, ITGB1BP1, STAB1*, *PLA2G4A*, *STIM2, VCL, DCLRE1B* and *USP20* (*P* < 0.05 for all cases, OR:2.31–3.88, Additional file 2: Table 2).

### Validation of survival-related genes

**Fig. 3:**
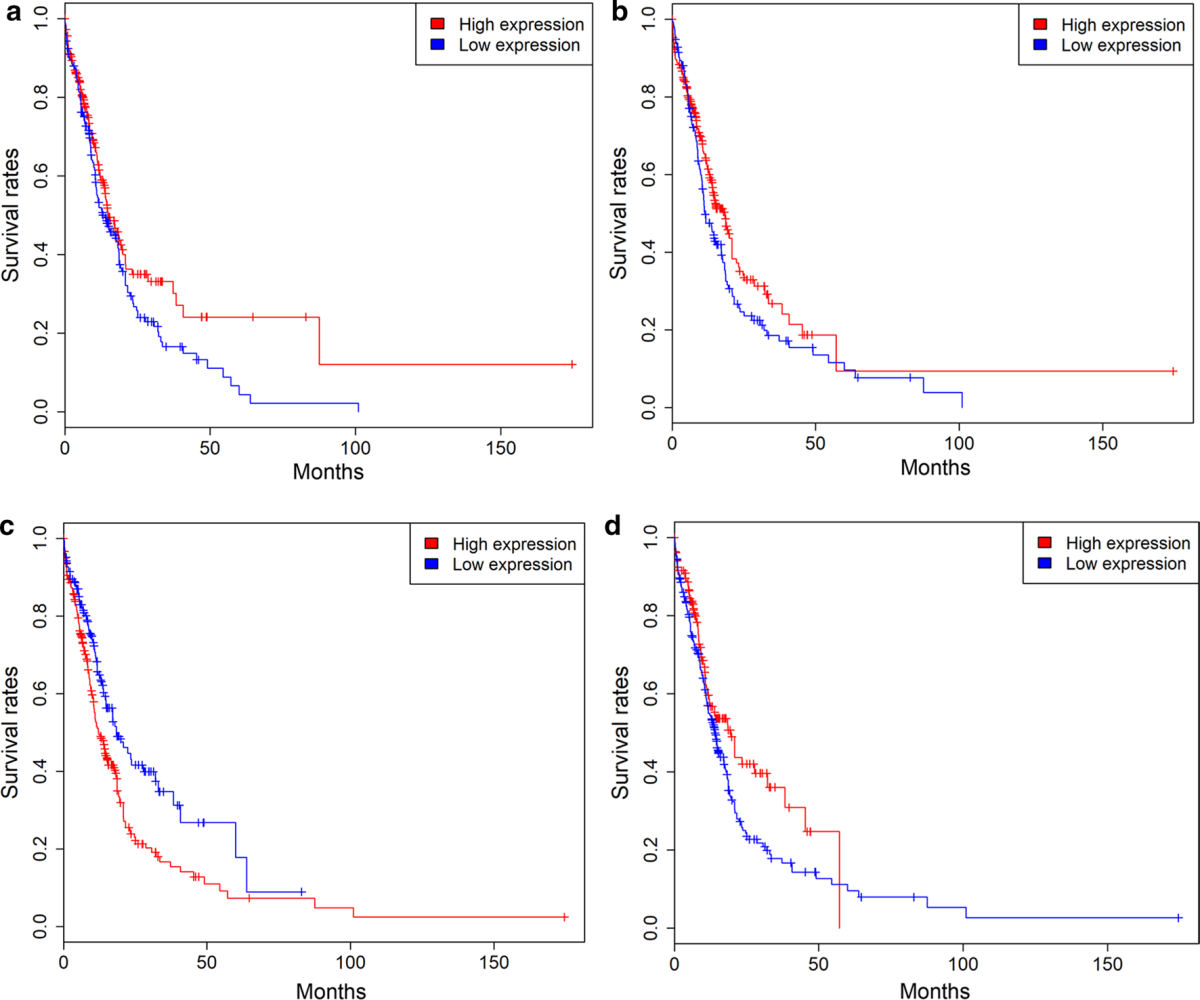
Kaplan–Meier survival analysis of patients’ OS with *VSTM1* (**a**), *FNDC3B* (**b**), *PLA2G4A* (**c**) and *CALR* (**d**) expression levels in 405 AML patients of the OHSU dataset

The clinical characteristics of AML patients in the OHSU cohort are presented in Additional file 2: Table 4. Kaplan–Meier survival analysis suggested that patients with high expression of 55 genes showed a favourable prognosis than those with low expression, such as *FNDC3B, VSTM1* and *CALR*. While, AML patients with high *IL15RA, VCL* and *PLA2G4A* expression had a poor prognosis than those with low *IL15RA, VCL* and *PLA2G4A* expression (*P* < 0.05 for all cases, log rank test, Fig. 3, Additional file 2: Table 5 and Additional file 1: Figure 3). Then, logistic regression model was applied between patients’ OS and 48 gene expression levels and the survival-related features, including age, cytogenetic risk, chemotherapy, bone marrow transplant, targeted therapy. 37 genes were demonstrated to be protective genes, such as *FNDC3B, VSTM1* and *CALR* (*P* < 0.05 for all cases, OR 0.29–0.34), while *VCL* and *PLA2G4A* was confirmed to be risk genes (*P* = 0.01, OR 1.99, *P* < 0.001, OR: 2.85, respectively, Additional file 2: Table 5 and Additional file 1: Figure 3).

### Unsupervised hierarchical clustering analysis

**Fig. 4:**
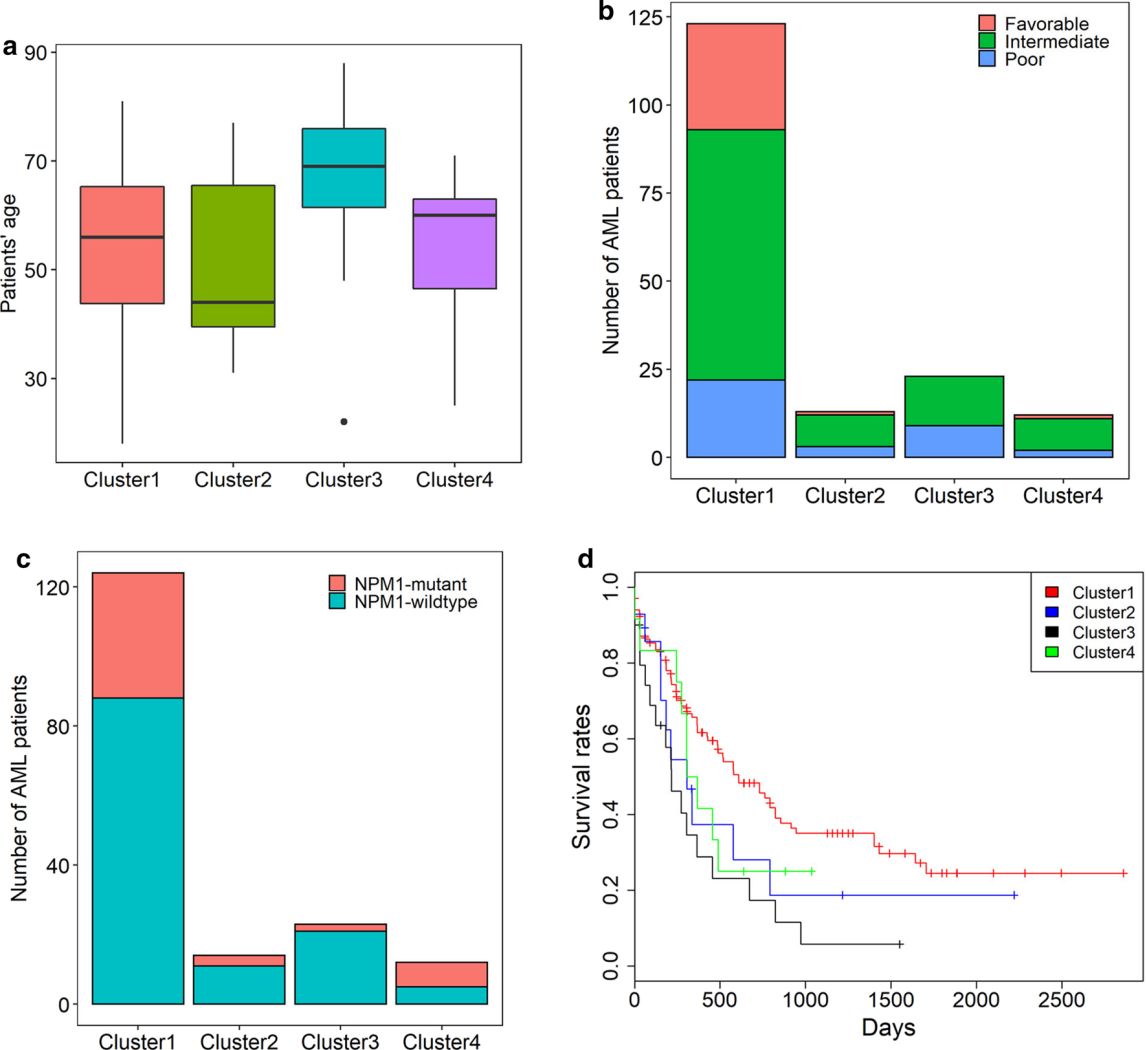
Differences in patients’ age (**a**), cytogenetic risk (**b**), *NPM1* mutation (**c**), and OS (**d**) were compared among the four clusters of AML patients (1–4) in the TCGA dataset

To build a panel of prognostic biomarkers to accurately evaluate the prognosis of AML patients, we selected the top five genes most significantly associated with patients’ OS. *FNDC3B, VSTM1, GOLGA3* and *CALR* showed the smallest four OR values and *P* values among the protective genes, *PLA2G4A* had the largest OR and smallest *P* value among the risk genes in the validation cohort. Therefore, these five genes were included in the gene panel for further survival analysis. Hierarchical clustering analysis of the five genes revealed four subgroups of AML patients (Additional file 1: Figure 4). The cluster1 AML patients showed younger age, lower cytogenetics risk, higher frequency of *NPM1* mutations and better OS than cluster3 patients (*P* values < 0.05 for all cases, Wilcoxon sum rank test, fisher exact test or log-rank test, Fig. 4 and Additional file 2: Table 6). The hierarchical clustering of the five genes also uncovered four subgroups of AML patients in the OHSU dataset (Additional file 1: Figure 5). Cluster1 tumors exhibited lower cytogenetics risk than those in cluster 3 or 4, lower frequency of *NPM1* mutations than cluster2 tumors, lower frequency of FLT3-ITD mutations than cluster 2 or 4 tumors and better OS than cluster 2, 3 or 4 tumors (*P* values < 0.05 for all cases, Wilcoxon sum rank test, fisher exact test or log-rank test, Additional file 1: Figure 6 and Additional file 2: Table 7).

### Risk score is a risk factor for overall survival in AML

**Fig. 5:**
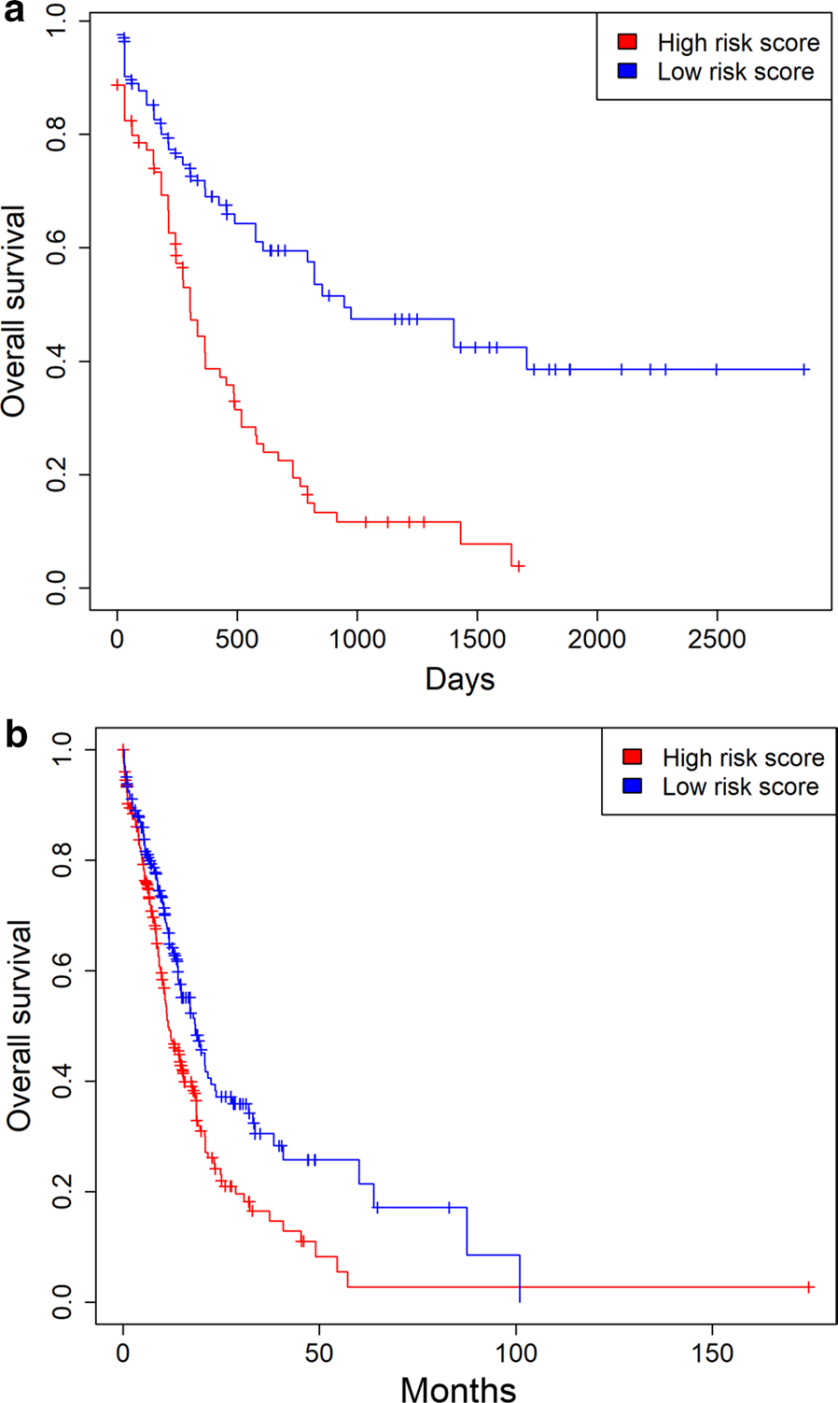
Risk score is a negative prognostic factor. **a** High risk score is associated with a poor prognosis in the TCGA dataset. **b** High risk score is associated with a poor prognosis in the OHSU dataset

We established the risk score model by a linear combination of the five genes *FNDC3B, VSTM1, PLA2G4A*, *GOLGA3* and *CALR* using the coefficients generated from the logistic regression models. Risk score = 0.32 × expression of *CALR* + 0.39 × expression of *VSTM1* + 3.88 × expression of *PLA2G4A* + 0.25 × expression of *GOLGA3* + 0.44 × expression of *FNDC3B* in the TCGA dataset. Kaplan–Meier survival analysis exhibited the risk score was negatively associated with OS of AML patients in the TCGA dataset (*P* < 0.05, log rank test and Fig. 5). The logistic regression model analysis validated that risk score was significantly associated with inferior OS following adjustment of survival-related features (*P* < 0.05 for all cases, Table2 and Fig. 5). To validate the findings above, risk score was calculated following the formula: risk score = 0.34 × expression of *CALR* + 0.32 × expression of *VSTM1* + 2.85 × expression of *PLA2G4A* + 0.35 × expression of *GOLGA3* + 0.29 × expression of *FNDC3B*. The negative correlation between OS and risk score was confirmed in the OHSU cohort (Table 2 and Fig. 5).

**Table 2:** Multivariate analyses between OS and the risk score in the TCGA and OHSU datasets

TCGA dataset				OHSU dataset			
Clinical feature	OR	95% CI	*P* value	Clinical feature	OR	95% CI	*P* value
Age	1.03	1.01–1.06	< 0.01	Age	1.04	1.02–1.05	< 0.001
Cytogenetic risk	1.71	0.97–3.1	0.07	Cytogenetic risk	0.97	0.74–1.28	0.85
Risk score	3.88	1.84–8.46	< 0.001	Chemotherapy	0.27	0.01–1.47	0.22
				Transplant	0.38	0.23–0.63	< 0.001
				Targeted therapy	2.87	1.51–5.71	< 0.002
				Risk score	2.37	1.49–3.81	< 0.001

*OR* odds ratio, *CI* confidence interval

## Discussion

WGCNA is a common computational tool to develop co-expression network and to identify the co-expression modules. Genes in the same module were regarded as functionally relevant. Thus, the application of WGCNA analysis enables the identification of clinical trait-associated modules which might become potentially prognostic and therapeutic targets [9]. In this study, 63 co-expression modules were detected by the WGCNA method using RNA-seq expression data of 18,366 genes from 173 AML samples. We identified 59/63 co-expression modules showed significant correlation with clinical traits. The OSAM1 module showed significantly negative correlation with age, cytogenetic risk, PBMBC, *DNMT3A* mutation and *NPM1* mutation and OS. The OSAM2 module was negatively associated with FAB subtypes and OS. GO enrichment analysis suggested that genes in the OSAM1 module were significantly enriched in 53 GO terms, such as negative regulation of signaling, regulation of cell communication, negative regulation of developmental process. Moreover, the genes in the OSAM1 module were over-represented in the KEGG pathway of protein processing in endoplasmic reticulum. Thus, we speculate that the OSAM1 and OSAM2 modules play a pivotal role in the overall survival of AML patients.FLT3-ITD frequently occurs in AML patients and indicates an inferior prognosis in AML [21]. There are 200 and 672 AML samples in the TCGA and OHSU datasets respectively, however, only 173 and 451 patients had somatic mutation and RNA-seq expression data. 173 AML patients in the TCGA cohort and 405 patients in the OHSU cohort were included in the study. Owing to the lack of FLT3-IDT information in the TCGA dataset, we analyzed the association between the FLT3 mutation and OS in the 173 patients with RNA-seq data and the 200 AML patients. However, no significant correlation was observed between the FLT3 mutation and overall survival (Additional file 2: Table 8). In the OHSU cohort, the FLT3-IDT mutation was indicative of poor prognosis in the 672 AML patients. However, the association was not statistically significant in our study (Additional file 2: Table 9). Therefore, the difference of our results and previous publications on the association of FLT3-IDT mutation with overall survival is probably caused by the selection of different cohorts of AML patients.

We analyzed the associations between 580 genes in the OSAM1 and OSAM2 modules and AML patients’ OS in the TCGA and OHSU datasets using many statistical methods and identified set of genes was significantly associated with OS in AML patients, such as *FNDC3B, VSTM1, PLA2G4A*, *GOLGA3* and *CALR*. The *PLA2G4A* gene encodes a member of the cytosolic phospholipase A2 group IV family which plays an important role the regulation of hemodynamics, inflammatory responses and other intracellular pathways [22]. The expression of *PLA2G4A* is up-regulated in a wide range of cancer types [23–26]. *PLA2G4A* depletion significantly repressed cellular proliferation in glioblastoma, lung cancer and colon cancer [23, 25, 26]. These results demonstrate *PLA2G4A* may play an oncogenic role in cancers. Another gene, *CALR*, has been involved in calcium retention and protein folding, as well as in immune responses [27]. In line with the finding in our study, *CALR* exposure by malignant blasts is correlated with robust anticancer immunity and superior OS in AML patients [28]. Activation of unfolded protein response, including *CALR*, is associated to more favorable clinical outcome and lower relapse rate [29]. These studies suggest *CALR* is a positive prognostic biomarker for AML patients.

High *FNDC3B, VSTM1, GOLGA3* and *CALR* expression and low *PLA2G4A* expression were indicative of decreased mortality of AML patients. Among the four subgroups of AML patients identified by hierarchical clustering analysis, the cluster1 AML patients showed younger age, lower cytogenetics risk, higher frequency of *NPM1* mutations and more favourable OS than cluster3 patients. Therefore, expression analysis of the gene panel might be clinically useful in the future. AML patients exhibiting low *FNDC3B, VSTM1, GOLGA3* and *CALR* expression or high *PLA2G4A* expression are expected to have poor clinical outcome. Therefore, these patients may need more aggressive therapies or more frequent follow-up.

Furthermore, we developed a risk score based on the linear combination of the five gene expression values. The risk score effectively stratifies AML patients with two distinct risk groups with significant different prognosis. Recent studies have reported a four-gene LincRNA expression signature (LINC4) and a 17-gene stemness score (LSC17) to predict risk in AML patients [30, 31]. Our risk score, LINC4 and LSC17, have all been tested on the TCGA dataset. Though the prognostic difference of subgroups of AML patients stratified by the three prognostication scores all were statistically significant, our risk showed higher OR value (3.88) than the OR values of LINC4 (2.22) and LSC17 (2.62), suggesting the risk score might have more predictive power for overall survival in AML. Moreover, the LSC17 score requires quantification of expression of 17 genes. Therefore, implementing the LSC17 risk classification might cause more experimental workload and higher cost than the LINC4 and our risk score. Lastly, the five genes may become druggable targets for AML patients. For instance, depletion of *PLA2G4A* caused significant decrease in cellular proliferation in glioblastoma, lung cancer and colon cancer cells [23, 25, 26].

## Conclusion

In conclusion, the OSAM1 and OSAM2 modules were the most critical modules in the OS of AML patients. The five gene panel comprising *FNDC3B, VSTM1, PLA2G4A*, *GOLGA3* and *CALR* and risk score may function as potential prognostic biomarkers for AML, which also needs much further research.

## Supplementary Information

**Additional file 1: **The supplementary figures which support the findings of this study.

**Additional file 2: **The supplementary tables which support the findings of this study.

## Data Availability

Raw data of the TCGA cohort are the normalized read counts (RNA-seq) data of 18379 genes of 173 AML patients and their clinical data were publicly available at https://figshare.com/s/7c683384c6e2add08262 (figshare ID: 13585235). The gene expression and clinical data of 405 AML patients (downloaded from the OHSU dataset) used for the validation of survival analysis in our study were publicly available at https://figshare.com/s/7c683384c6e2add08262 (figshare ID: 13585235).

## Abbreviations

AML: Acute myeloid leukemia
TCGA: The Cancer Genome Altas
OHSU: The Oregon Health & Science University
OS: Overall survival
GO: Gene ontology
KEGG: Kyoto Encyclopedia of Genes and Genomes
WGCNA: Weighted correlation network analysis
FAB: French-American-British
PBMBC: Percent of bone marrow blast cells
FDR: False discovery rate
AUC: Area under curve
OR: Odds ratio
OSAM1: Overall survival-associated module 1
OSAM2: Overall survival-associated module 2
