# Supplementary material for: Novel prognostic genes and subclasses of acute myeloid leukemia revealed by survival analysis of gene expression data
Source: BMC Med Genomics. 2021 Feb 3;14:39. doi: 10.1186/s12920-021-00888-0 (PMC7860023; doi:10.1186/s12920-021-00888-0)
Supplement: Supplementary file 1 — Additional file 1: The supplementary figures which support the findings of this study. [file 12920_2021_888_MOESM1_ESM.docx]

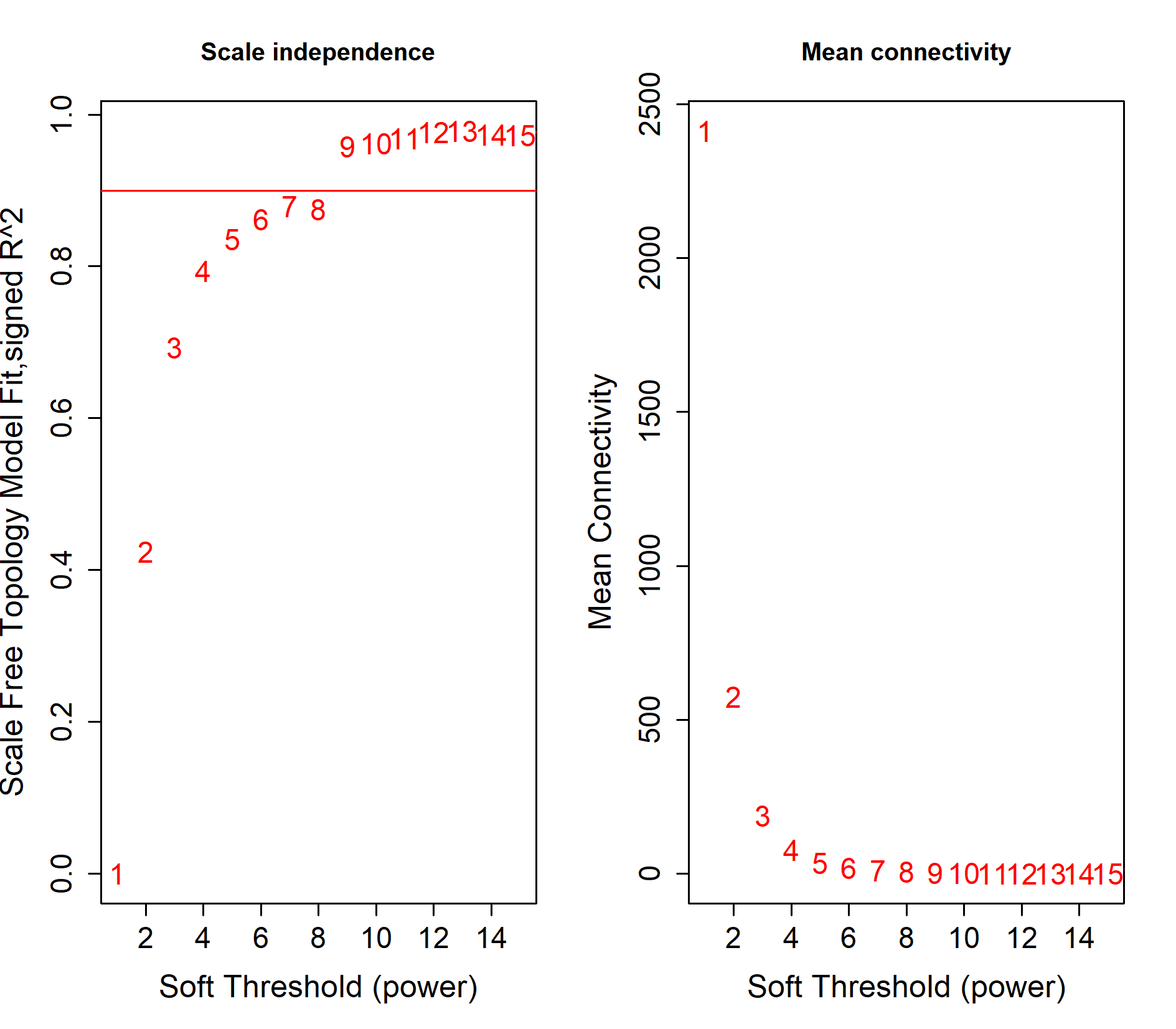


Supplementary Figure1. Analysis of network topology for various soft-thresholding powers. The left panel shows the scale-free fit index (y-axis) as a function of the soft-thresholding power (x-axis). The right panel displays the mean connectivity (degree, y-axis) as a function of the soft-thresholding power (x-axis).


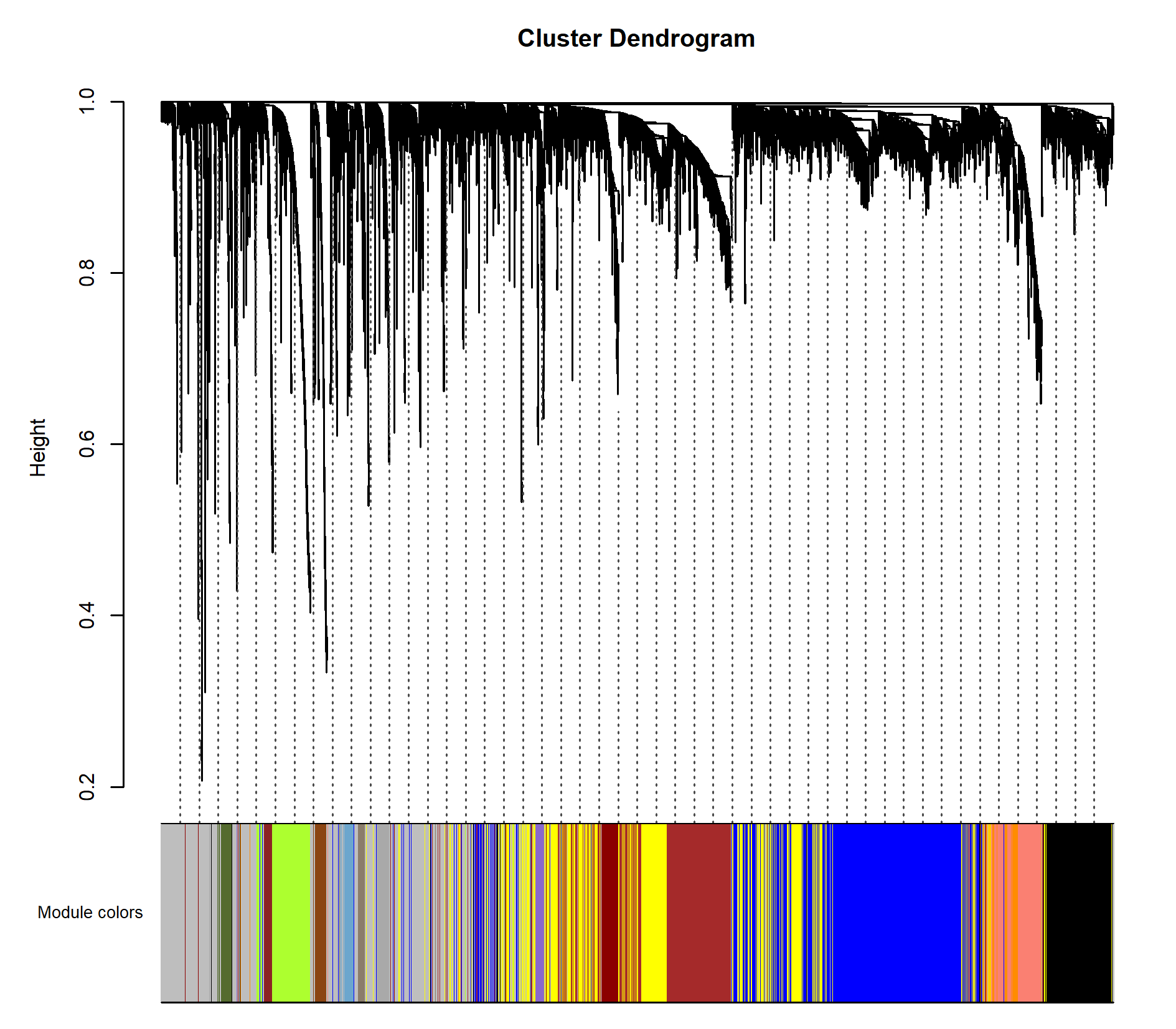


Supplementary Figure2. Hierarchical clustering dendrograms of 18366 genes detected 65 co-expression modules which were shown in colors.


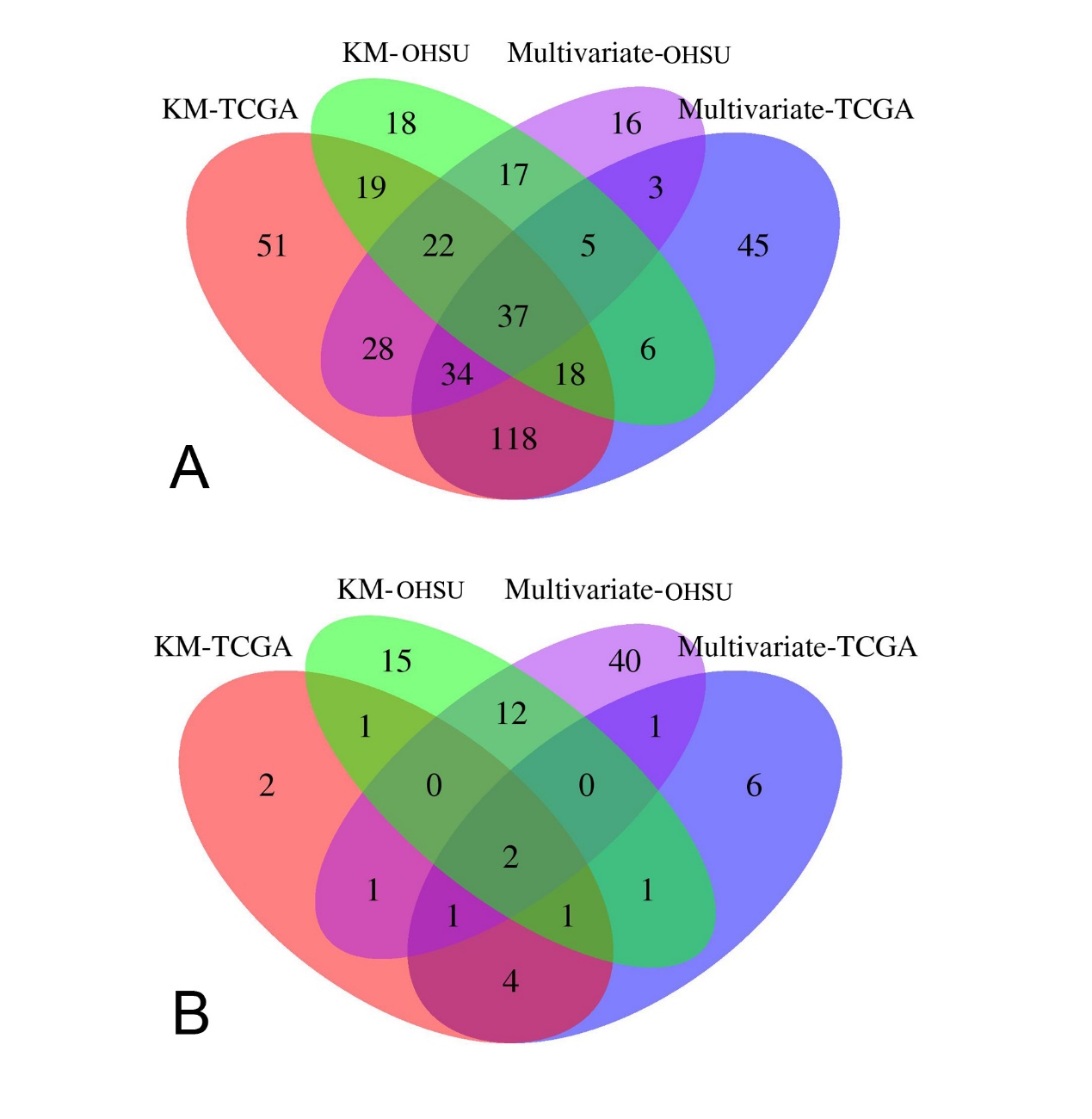
Supplementary Figure3. The overlap of prognosis-associated genes between the TCGA and OHSU datasets. A. The overlap of protective genes between the TCGA and OHSU datasets. B. The overlap of risk genes between the TCGA and OHSU datasets.


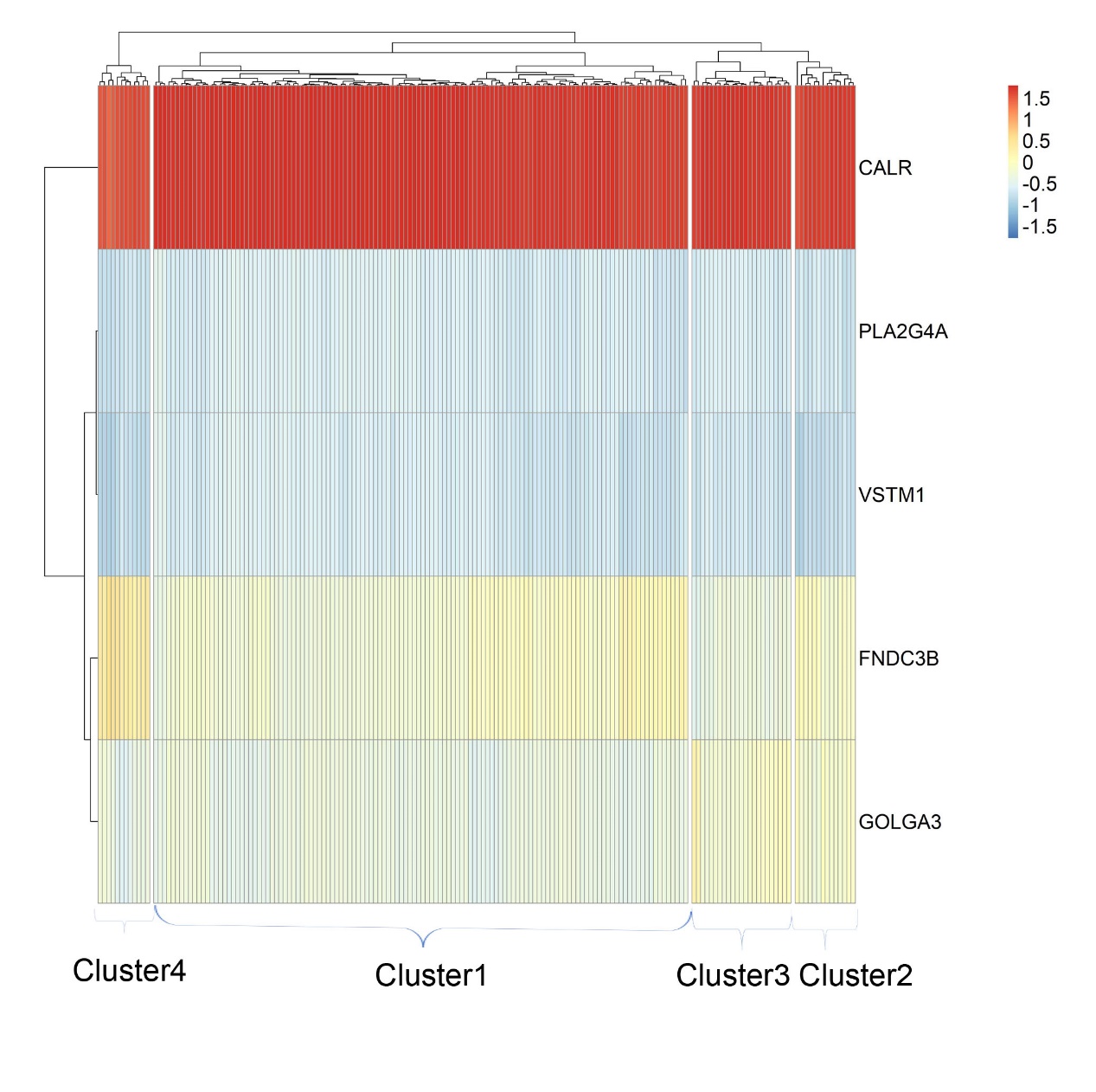


Supplementary Figure4. Unsupervised hierarchical clustering of the five gene panel revealed four subgroups of AML patients in the TCGA dataset.


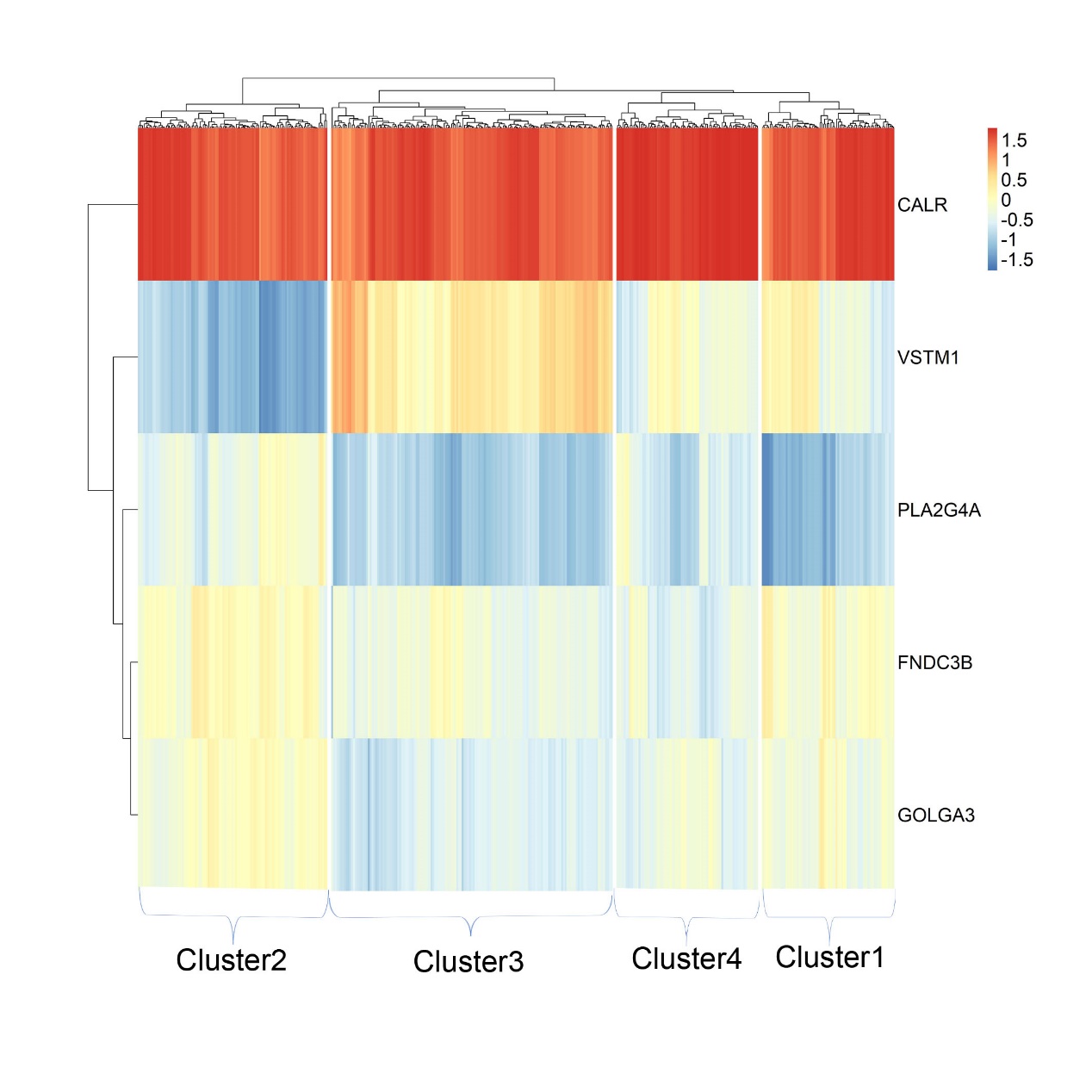


Supplementary Figure5. Unsupervised hierarchical clustering of the five gene panel revealed four subgroups of AML patients in the validation dataset.


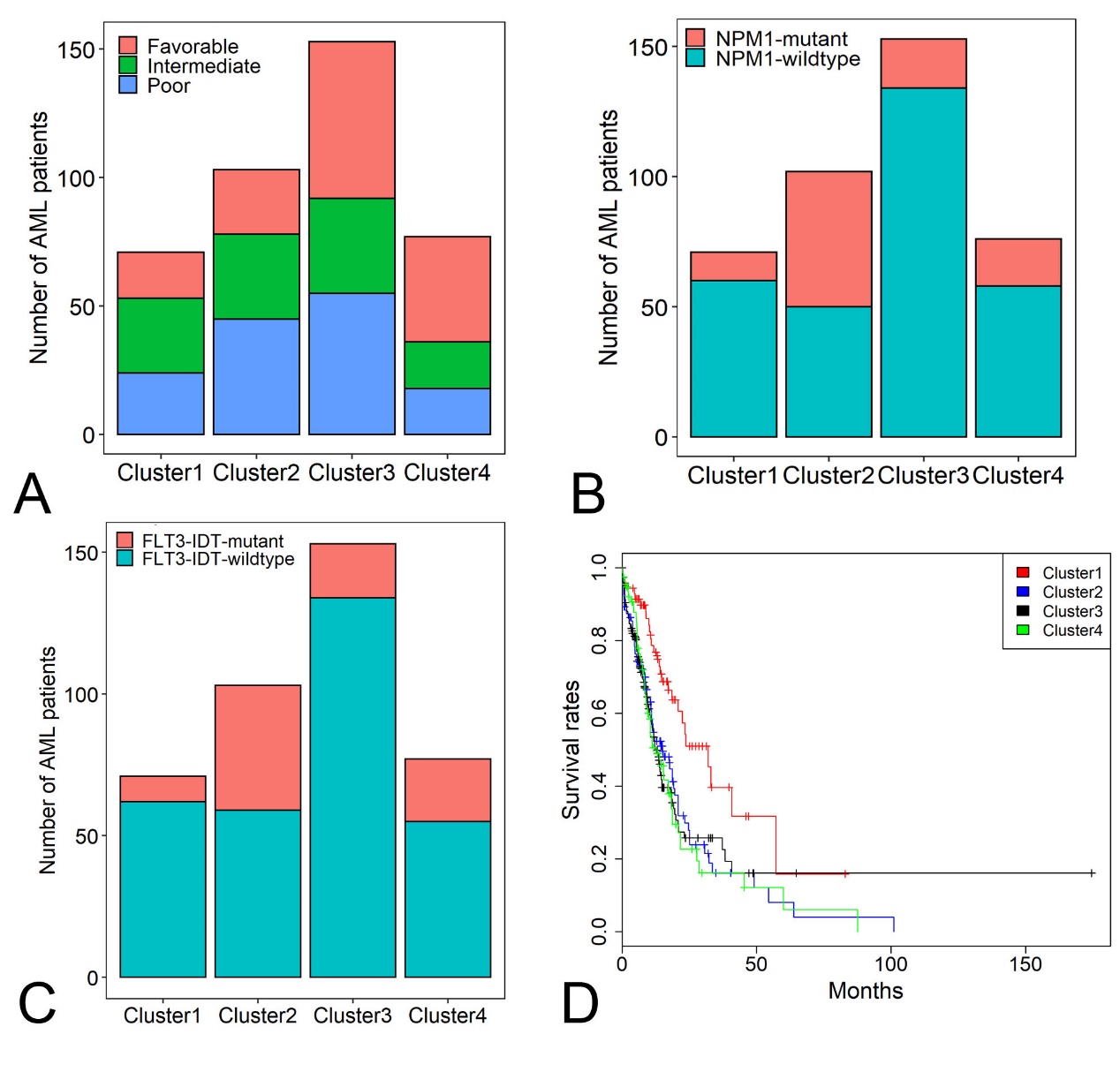


Supplementary Figure6. Differences in cytogenetic risk (A), *NPM1* mutation (B), FLT3-ITD mutation (C), and OS (D) were compared among the four clusters of AML patients (1–4) in the validation dataset.
