# Supplementary material for: Novel prognostic genes and subclasses of acute myeloid leukemia revealed by survival analysis of gene expression data
Source: BMC Med Genomics. 2021 Feb 3;14:39. doi: 10.1186/s12920-021-00888-0 (PMC7860023; doi:10.1186/s12920-021-00888-0)
Supplement: Supplementary file 2 — Additional file 2: The supplementary tables which support the findings of this study. [file 12920_2021_888_MOESM2_ESM.docx]

Supplementary Table1. the co-expression modules in the WGCNA network

| Module | Number of genes | Correlation with clinical traits |
| --- | --- | --- |
| lightcyan1 | 75 | BMBC |
| antiquewhite4 | 35 | None |
| orangered4 | 78 | Age, BMBC,Cytogenetic risk,FLT3 mutation |
| floralwhite | 71 | BMBC,DNMT3A mutation, FAB, neoadjuvant treatment |
| darkturquoise | 136 | Age, BMBC,DNMT3A mutation, FAB, neoadjuvant treatment |
| brown | 1070 | Age, BMBC, FAB |
| yellow | 1067 | Age, BMBC,DNMT3A mutation, NP1 mutation, FAB, neoadjuvant treatment |
| sienna3 | 84 | BMBC,NP1 mutation, neoadjuvant treatment |
| plum1 | 78 | BMBC, gender,NP1 mutation,FLT3 mutation, FAB, neoadjuvant treatment |
| thistle1 | 64 | BMBC,Cytogenetic risk,DNMT3A mutation,NP1 mutation,CEBPA mutation,FLT3 mutation, neoadjuvant treatment |
| darkmagenta | 85 | BMBC, IDH1 mutation, FAB |
| green | 548 | IDH1 mutation,NP1 mutation |
| darkgrey | 125 | FAB, neoadjuvant treatment |
| ivory | 73 | Cytogenetic risk,NP1 mutation |
| lightgreen | 186 | Age, NP1 mutation, FAB |
| lavenderblush3 | 49 | BMBC,Cytogenetic risk, FAB |
| turquoise | 2999 | None |
| thistle2 | 65 | BMBC,Cytogenetic risk |
| palevioletred3 | 56 | Cytogenetic risk, NP1 mutation |
| pink | 429 | Age, BMBC,Cytogenetic risk,OS,DNMT3A mutation, NP1 mutation |
| darkgreen | 148 | IDH2 mutation |
| cyan | 244 | Gender |
| skyblue | 112 | Gender |
| darkolivegreen | 90 | BMBC, FAB |
| orange | 117 | BMBC, FAB |
| lightpink4 | 51 | BMBC, FAB |
| brown4 | 70 | BMBC,Cytogenetic risk,DNMT3A mutation, FAB |
| midnightblue | 217 | FAB |
| navajowhite2 | 56 | IDH1 mutation |
| bisque4 | 68 | Age,Cytogenetic risk,NP1 mutation |
| violet | 91 | BMBC,Cytogenetic risk,NP1 mutation |
| steelblue | 94 | Cytogenetic risk, FAB |
| greenyellow | 325 | FAB |
| plum2 | 65 | None |
| blue | 1497 | BMBC, FAB |
| paleturquoise | 93 | Cytogenetic risk, gender, FAB |
| maroon | 53 | Cytogenetic risk, gender, FAB |
| red | 539 | None |
| darkorange | 116 | BMBC, NP1 mutation,CEBPA mutation |
| grey60 | 194 | FLT3 mutation |
| coral2 | 34 | None |
| salmon4 | 58 | Age |
| darkslateblue | 66 | Cytogenetic risk,NP1 mutation |
| lightsteelblue1 | 77 | NP1 mutation |
| mediumpurple3 | 78 | IDH2 mutation,NP1 mutation |
| darkseagreen4 | 43 | Age, NP1 mutation,FLT3 mutation |
| skyblue3 | 80 | Age,Cytogenetic risk,IDH2 mutation,NP1 mutation,FLT3 mutation, FAB, neoadjuvant treatment |
| yellowgreen | 83 | FAB |
| saddlebrown | 99 | Cytogenetic risk,NP1 mutation, FAB |
| honeydew1 | 45 | Age,Cytogenetic risk,NP1 mutation |
| darkred | 151 | OS, FAB |
| lightyellow | 177 | Cytogenetic risk,NP1 mutation,FLT3 mutation, FAB |
| magenta | 417 | None |
| coral1 | 43 | None |
| royalblue | 163 | None |
| salmon | 261 | Age, BMBC,NP1 mutation,FLT3 mutation, neoadjuvant treatment |
| white | 113 | Age, BMBC |
| darkorange2 | 71 | Age, NP1 mutation |
| black | 495 | Cytogenetic risk |
| lightcyan | 208 | BMBC,Cytogenetic risk,FLT3 mutation, neoadjuvant treatment |
| purple | 365 | BMBC,FAB |
| tan | 305 | BMBC, NP1 mutation,FLT3 mutation, FAB, neoadjuvant treatment |
| grey | 3321 | BMBC,Cytogenetic risk,FLT3 mutation, FAB |

Notably, percent of bone marrow blast cells: PBMBC

Supplementary Table2. Kaplan-Meier survival analysis and multivariate analyses between gene expression and OS of AML patients

in the TCGA dataset

|  | Kaplan-Meier survival analysis | | | Multivariate analysis | | | |
| --- | --- | --- | --- | --- | --- | --- | --- |
| Gene | chisq | P value | Cutoff | OR | 2.50%CI | 97.50%CI | P value |
| ABCB10 | 5.27 | 0.02 | 1207.39 | 0.58 | 0.29 | 1.17 | 0.13 |
| ACCN2 | 6.68 | 0.01 | 32.83 | 0.56 | 0.26 | 1.22 | 0.14 |
| ACCS | 8.55 | 0.00 | 1077.26 | 0.41 | 0.19 | 0.84 | 0.02 |
| ACSM1 | 8.42 | 0.00 | 25.93 | 0.46 | 0.22 | 0.95 | 0.03 |
| ADAMTS15 | 7.94 | 0.00 | 2.20 | 0.48 | 0.23 | 1.04 | 0.06 |
| ADAMTS18 | 5.59 | 0.02 | 133.53 | 0.34 | 0.08 | 1.25 | 0.12 |
| AFF2 | 12.80 | 0.00 | 198.88 | 0.48 | 0.20 | 1.10 | 0.09 |
| AGAP4 | 7.52 | 0.01 | 1046.28 | 0.25 | 0.08 | 0.73 | 0.01 |
| AGRN | 8.43 | 0.00 | 821.16 | 0.28 | 0.13 | 0.59 | 0.00 |
| AKAP12 | 9.07 | 0.00 | 32.63 | 0.51 | 0.25 | 1.03 | 0.06 |
| AMIGO1 | 4.95 | 0.03 | 46.60 | 0.52 | 0.26 | 1.05 | 0.07 |
| ANKFY1 | 9.82 | 0.00 | 3891.06 | 0.42 | 0.12 | 1.32 | 0.14 |
| ANKLE2 | 8.92 | 0.00 | 2785.13 | 0.69 | 0.32 | 1.53 | 0.35 |
| ANKRD27 | 11.50 | 0.00 | 3270.76 | 0.43 | 0.19 | 0.97 | 0.04 |
| ANKRD35 | 15.61 | 0.00 | 103.86 | 0.11 | 0.03 | 0.35 | 0.00 |
| ARHGAP10 | 8.77 | 0.00 | 200.43 | 0.64 | 0.32 | 1.30 | 0.22 |
| ARMCX1 | 4.21 | 0.04 | 130.35 | 0.51 | 0.24 | 1.06 | 0.08 |
| ARMCX3 | 11.11 | 0.00 | 1266.43 | 0.09 | 0.00 | 0.58 | 0.03 |
| ASNS | 9.62 | 0.00 | 981.76 | 0.14 | 0.03 | 0.53 | 0.01 |
| ASS1 | 6.32 | 0.01 | 25.60 | 0.47 | 0.20 | 1.09 | 0.08 |
| ATP12A | 10.09 | 0.00 | 1.03 | 0.43 | 0.21 | 0.89 | 0.02 |
| ATP1B1 | 11.80 | 0.00 | 686.06 | 0.19 | 0.08 | 0.43 | 0.00 |
| ATP6V1C2 | 8.68 | 0.00 | 194.49 | 0.37 | 0.10 | 1.27 | 0.12 |
| AUTS2 | 12.94 | 0.00 | 463.70 | 0.33 | 0.16 | 0.67 | 0.00 |
| AVEN | 4.86 | 0.03 | 175.12 | 0.61 | 0.29 | 1.26 | 0.18 |
| AZU1 | 7.50 | 0.01 | 3598.27 | 0.46 | 0.22 | 0.95 | 0.04 |
| B3GNT1 | 11.60 | 0.00 | 277.49 | 0.08 | 0.00 | 0.49 | 0.02 |
| BMS1P5 | 5.52 | 0.02 | 944.47 | 0.58 | 0.28 | 1.22 | 0.15 |
| BZRAP1 | 10.43 | 0.00 | 3336.81 | 0.43 | 0.21 | 0.87 | 0.02 |
| C14orf174 | 6.29 | 0.01 | 43.77 | 0.40 | 0.18 | 0.89 | 0.02 |
| C14orf64 | 11.84 | 0.00 | 106.02 | 0.24 | 0.09 | 0.62 | 0.00 |
| C16orf3 | 10.31 | 0.00 | 10.05 | 0.36 | 0.17 | 0.78 | 0.01 |
| C17orf85 | 4.71 | 0.03 | 1388.76 | 0.54 | 0.26 | 1.12 | 0.10 |
| C20orf3 | 4.74 | 0.03 | 1597.20 | 0.59 | 0.29 | 1.19 | 0.14 |
| C2orf65 | 10.94 | 0.00 | 77.30 | 0.41 | 0.20 | 0.85 | 0.02 |
| C3orf33 | 11.19 | 0.00 | 70.14 | 0.53 | 0.26 | 1.09 | 0.08 |
| C4orf38 | 9.07 | 0.00 | 1.67 | 0.49 | 0.22 | 1.06 | 0.07 |
| C9orf43 | 14.72 | 0.00 | 241.69 | 0.17 | 0.05 | 0.50 | 0.00 |
| CALR | 10.21 | 0.00 | 24461.78 | 0.32 | 0.13 | 0.76 | 0.01 |
| CAMK2G | 7.41 | 0.01 | 2203.42 | 0.59 | 0.23 | 1.56 | 0.29 |
| CCRN4L | 14.20 | 0.00 | 1095.45 | 0.11 | 0.02 | 0.38 | 0.00 |
| CD3D | 4.69 | 0.03 | 405.13 | 0.48 | 0.17 | 1.34 | 0.16 |
| CDH26 | 8.61 | 0.00 | 89.03 | 0.46 | 0.21 | 1.01 | 0.05 |
| CDON | 7.76 | 0.01 | 36.61 | 0.45 | 0.18 | 1.11 | 0.08 |
| CDR2 | 9.23 | 0.00 | 317.04 | 0.72 | 0.32 | 1.62 | 0.41 |
| CERCAM | 4.45 | 0.03 | 475.29 | 0.53 | 0.26 | 1.07 | 0.07 |
| CHRM5 | 5.75 | 0.02 | 46.39 | 0.51 | 0.15 | 1.73 | 0.28 |
| CHRNB1 | 5.48 | 0.02 | 139.87 | 0.59 | 0.29 | 1.19 | 0.14 |
| CITED2 | 9.59 | 0.00 | 4118.32 | 0.23 | 0.10 | 0.50 | 0.00 |
| CLDN12 | 7.42 | 0.01 | 128.85 | 0.55 | 0.27 | 1.12 | 0.11 |
| CLTCL1 | 6.55 | 0.01 | 1305.93 | 0.47 | 0.19 | 1.16 | 0.10 |
| CORO2B | 6.18 | 0.01 | 26.15 | 0.58 | 0.28 | 1.20 | 0.14 |
| CPA3 | 6.97 | 0.01 | 7644.76 | 0.28 | 0.11 | 0.70 | 0.01 |
| CPA6 | 5.34 | 0.02 | 80.00 | 0.28 | 0.09 | 0.82 | 0.02 |
| CSF3R | 7.33 | 0.01 | 9659.39 | 0.35 | 0.16 | 0.75 | 0.01 |
| CTSF | 4.25 | 0.04 | 33.51 | 1.24 | 0.51 | 2.96 | 0.63 |
| CTSW | 9.32 | 0.00 | 10527.79 | 0.18 | 0.02 | 0.88 | 0.05 |
| CYP2E1 | 9.21 | 0.00 | 64.68 | 0.43 | 0.20 | 0.92 | 0.03 |
| CYP3A7 | 5.18 | 0.02 | 0.89 | 0.47 | 0.22 | 0.96 | 0.04 |
| CYP4F11 | 5.39 | 0.02 | 6.21 | 0.83 | 0.39 | 1.79 | 0.63 |
| CYTL1 | 8.55 | 0.00 | 1266.11 | 0.56 | 0.26 | 1.21 | 0.14 |
| DCLK2 | 6.13 | 0.01 | 34.35 | 0.42 | 0.20 | 0.90 | 0.03 |
| DDIT4L | 7.89 | 0.00 | 119.58 | 0.35 | 0.12 | 0.97 | 0.05 |
| DDOST | 8.92 | 0.00 | 5788.12 | 0.40 | 0.17 | 0.94 | 0.03 |
| DHRS7 | 8.03 | 0.00 | 1248.42 | 0.27 | 0.10 | 0.71 | 0.01 |
| DLG5 | 4.17 | 0.04 | 634.20 | 0.34 | 0.15 | 0.75 | 0.01 |
| DLL3 | 15.29 | 0.00 | 5.46 | 0.32 | 0.12 | 0.81 | 0.02 |
| DNAJB11 | 12.90 | 0.00 | 1185.29 | 0.30 | 0.10 | 0.88 | 0.03 |
| DNM1 | 9.32 | 0.00 | 758.90 | 0.36 | 0.17 | 0.75 | 0.01 |
| DOC2B | 13.36 | 0.00 | 3.38 | 0.46 | 0.21 | 1.03 | 0.06 |
| EEPD1 | 3.89 | 0.05 | 347.43 | 0.56 | 0.27 | 1.14 | 0.11 |
| EFNB3 | 9.59 | 0.00 | 22.95 | 0.30 | 0.10 | 0.85 | 0.03 |
| ELANE | 13.61 | 0.00 | 5559.50 | 0.33 | 0.13 | 0.79 | 0.01 |
| ELL3 | 10.14 | 0.00 | 96.90 | 0.40 | 0.14 | 1.11 | 0.08 |
| EMILIN1 | 4.48 | 0.03 | 1153.14 | 0.46 | 0.17 | 1.22 | 0.12 |
| EPHA10 | 8.13 | 0.00 | 3.36 | 0.24 | 0.09 | 0.60 | 0.00 |
| EPHX1 | 7.88 | 0.00 | 241.83 | 0.29 | 0.13 | 0.61 | 0.00 |
| ERLIN1 | 9.70 | 0.00 | 3589.41 | 0.53 | 0.26 | 1.08 | 0.08 |
| EXT2 | 13.15 | 0.00 | 1559.38 | 0.52 | 0.24 | 1.15 | 0.10 |
| F2 | 6.73 | 0.01 | 5.43 | 0.41 | 0.18 | 0.94 | 0.03 |
| FAM101A | 7.69 | 0.01 | 8.08 | 0.59 | 0.29 | 1.18 | 0.14 |
| FAM125B | 6.37 | 0.01 | 526.94 | 0.56 | 0.27 | 1.11 | 0.10 |
| FAM198A | 10.57 | 0.00 | 61.57 | 0.27 | 0.07 | 0.86 | 0.03 |
| FAM19A5 | 8.62 | 0.00 | 1.82 | 0.39 | 0.19 | 0.79 | 0.01 |
| FAM41C | 7.04 | 0.01 | 81.08 | 0.45 | 0.13 | 1.49 | 0.19 |
| FAM45A | 10.45 | 0.00 | 1180.24 | 0.45 | 0.21 | 0.96 | 0.04 |
| FAM45B | 8.93 | 0.00 | 463.85 | 0.40 | 0.19 | 0.85 | 0.02 |
| FAM46C | 5.27 | 0.02 | 2251.16 | 0.32 | 0.14 | 0.71 | 0.01 |
| FBLN1 | 5.94 | 0.01 | 8.75 | 0.59 | 0.29 | 1.18 | 0.14 |
| FBN2 | 6.04 | 0.01 | 51.04 | 0.30 | 0.11 | 0.73 | 0.01 |
| FGF13 | 9.05 | 0.00 | 31.63 | 0.40 | 0.16 | 1.01 | 0.05 |
| FGFR1 | 10.76 | 0.00 | 1305.63 | 0.39 | 0.15 | 0.99 | 0.05 |
| FGFR3 | 3.91 | 0.05 | 46.96 | 0.46 | 0.22 | 0.94 | 0.03 |
| FLJ39609 | 10.15 | 0.00 | 2.10 | 0.25 | 0.11 | 0.56 | 0.00 |
| FNDC3B | 12.26 | 0.00 | 3922.68 | 0.44 | 0.22 | 0.90 | 0.02 |
| FSTL3 | 6.92 | 0.01 | 405.10 | 0.44 | 0.22 | 0.90 | 0.02 |
| GALNT2 | 4.00 | 0.05 | 2654.99 | 0.51 | 0.22 | 1.19 | 0.11 |
| GAS8 | 11.12 | 0.00 | 200.80 | 0.23 | 0.10 | 0.51 | 0.00 |
| GFI1 | 10.26 | 0.00 | 2109.06 | 0.49 | 0.23 | 1.04 | 0.06 |
| GFRA3 | 8.55 | 0.00 | 1.86 | 0.56 | 0.25 | 1.26 | 0.16 |
| GJA3 | 6.32 | 0.01 | 5.22 | 0.66 | 0.33 | 1.36 | 0.26 |
| GLT8D1 | 4.49 | 0.03 | 707.60 | 0.54 | 0.24 | 1.15 | 0.11 |
| GOLGA2 | 4.71 | 0.03 | 1031.37 | 0.33 | 0.14 | 0.79 | 0.01 |
| GOLGA3 | 7.95 | 0.00 | 3456.36 | 0.25 | 0.06 | 0.91 | 0.04 |
| GPR153 | 10.58 | 0.00 | 174.72 | 0.29 | 0.11 | 0.71 | 0.01 |
| GPT2 | 9.36 | 0.00 | 984.24 | 0.27 | 0.11 | 0.65 | 0.00 |
| GRASP | 4.10 | 0.04 | 99.35 | 0.61 | 0.28 | 1.35 | 0.21 |
| GSDMB | 4.74 | 0.03 | 225.19 | 0.42 | 0.20 | 0.90 | 0.03 |
| GSG1L | 5.08 | 0.02 | 10.28 | 0.65 | 0.26 | 1.62 | 0.35 |
| GSG1 | 11.24 | 0.00 | 7.00 | 0.33 | 0.11 | 0.95 | 0.04 |
| H2AFV | 18.69 | 0.00 | 4015.58 | 0.49 | 0.24 | 0.98 | 0.04 |
| H2AFY | 4.41 | 0.04 | 10786.26 | 0.72 | 0.35 | 1.50 | 0.38 |
| HGF | 10.38 | 0.00 | 1505.65 | 0.46 | 0.22 | 0.99 | 0.04 |
| HGSNAT | 6.37 | 0.01 | 3570.40 | 0.47 | 0.22 | 1.01 | 0.05 |
| HIPK1 | 5.67 | 0.02 | 5930.57 | 0.60 | 0.30 | 1.21 | 0.15 |
| HRASLS5 | 7.28 | 0.01 | 93.26 | 0.51 | 0.14 | 1.76 | 0.29 |
| HRH4 | 7.22 | 0.01 | 21.66 | 0.75 | 0.37 | 1.55 | 0.43 |
| HRNBP3 | 10.62 | 0.00 | 59.07 | 0.35 | 0.14 | 0.90 | 0.03 |
| HSDL1 | 14.88 | 0.00 | 669.35 | 0.34 | 0.12 | 0.92 | 0.04 |
| HSP90B1 | 14.97 | 0.00 | 18276.75 | 0.28 | 0.10 | 0.76 | 0.01 |
| HSP90B3P | 9.38 | 0.00 | 5.56 | 0.47 | 0.20 | 1.02 | 0.06 |
| HSPA4L | 7.97 | 0.00 | 134.06 | 0.32 | 0.13 | 0.79 | 0.01 |
| HYOU1 | 9.86 | 0.00 | 4546.28 | 0.40 | 0.14 | 1.08 | 0.07 |
| IFT52 | 5.15 | 0.02 | 439.73 | 0.59 | 0.29 | 1.21 | 0.15 |
| IGDCC4 | 11.49 | 0.00 | 4.76 | 0.46 | 0.23 | 0.93 | 0.03 |
| IGLON5 | 8.96 | 0.00 | 26.27 | 0.17 | 0.03 | 0.67 | 0.02 |
| IL15RA | 10.00 | 0.00 | 87.20 | 2.55 | 1.16 | 5.66 | 0.02 |
| INSR | 18.90 | 0.00 | 1306.68 | 0.31 | 0.12 | 0.73 | 0.01 |
| IRX3 | 13.51 | 0.00 | 538.50 | 0.15 | 0.04 | 0.44 | 0.00 |
| ITGB1BP1 | 7.68 | 0.01 | 637.29 | 2.98 | 1.39 | 6.76 | 0.01 |
| ITGB6 | 12.52 | 0.00 | 1.16 | 0.34 | 0.16 | 0.70 | 0.00 |
| KBTBD11 | 4.55 | 0.03 | 1418.97 | 0.48 | 0.23 | 0.98 | 0.05 |
| KIAA0195 | 10.60 | 0.00 | 3412.19 | 0.20 | 0.07 | 0.51 | 0.00 |
| KIAA0427 | 10.85 | 0.00 | 763.00 | 0.22 | 0.06 | 0.67 | 0.01 |
| KIAA0895 | 9.76 | 0.00 | 196.55 | 0.32 | 0.11 | 0.86 | 0.03 |
| KIF26A | 8.18 | 0.00 | 168.12 | 0.31 | 0.12 | 0.73 | 0.01 |
| KRT17 | 9.31 | 0.00 | 111.02 | 0.30 | 0.11 | 0.79 | 0.02 |
| LAMC3 | 11.05 | 0.00 | 63.60 | 0.22 | 0.06 | 0.71 | 0.01 |
| LGR4 | 15.20 | 0.00 | 68.38 | 0.22 | 0.10 | 0.46 | 0.00 |
| LHFP | 4.63 | 0.03 | 109.56 | 0.39 | 0.19 | 0.81 | 0.01 |
| LIN7A | 8.83 | 0.00 | 20.30 | 0.22 | 0.10 | 0.47 | 0.00 |
| LOC100130015 | 11.40 | 0.00 | 33.79 | 0.36 | 0.14 | 0.89 | 0.03 |
| LOC100130386 | 9.43 | 0.00 | 0.71 | 0.35 | 0.16 | 0.72 | 0.01 |
| LOC283663 | 6.68 | 0.01 | 445.95 | 0.47 | 0.22 | 0.95 | 0.04 |
| LOC441208 | 6.00 | 0.01 | 122.69 | 0.38 | 0.18 | 0.78 | 0.01 |
| LOC642826 | 8.73 | 0.00 | 483.21 | 0.43 | 0.20 | 0.91 | 0.03 |
| LOC729668 | 13.44 | 0.00 | 0.86 | 0.34 | 0.15 | 0.73 | 0.01 |
| LOC93622 | 10.83 | 0.00 | 322.11 | 0.33 | 0.14 | 0.77 | 0.01 |
| LOXL4 | 23.31 | 0.00 | 91.55 | 0.21 | 0.09 | 0.49 | 0.00 |
| LPO | 15.94 | 0.00 | 354.69 | 0.40 | 0.17 | 0.92 | 0.03 |
| LTK | 9.77 | 0.00 | 549.80 | 0.38 | 0.16 | 0.89 | 0.03 |
| MANF | 8.75 | 0.00 | 797.59 | 0.49 | 0.20 | 1.18 | 0.11 |
| MAVS | 3.98 | 0.05 | 7669.77 | 0.70 | 0.34 | 1.46 | 0.34 |
| MBTPS1 | 21.29 | 0.00 | 2605.91 | 0.37 | 0.18 | 0.77 | 0.01 |
| MEG3 | 4.88 | 0.03 | 4.67 | 0.34 | 0.16 | 0.68 | 0.00 |
| MEGF6 | 8.68 | 0.00 | 2189.34 | 0.31 | 0.15 | 0.64 | 0.00 |
| MICALL2 | 5.69 | 0.02 | 944.87 | 0.33 | 0.15 | 0.72 | 0.01 |
| MLEC | 18.29 | 0.00 | 7197.61 | 0.34 | 0.15 | 0.78 | 0.01 |
| MMP2 | 8.13 | 0.00 | 711.94 | 0.36 | 0.18 | 0.74 | 0.01 |
| MOSC2 | 4.29 | 0.04 | 56.95 | 0.57 | 0.28 | 1.18 | 0.13 |
| MPO | 14.01 | 0.00 | 98600.52 | 0.47 | 0.21 | 1.07 | 0.07 |
| MRAS | 4.58 | 0.03 | 88.49 | 0.51 | 0.23 | 1.14 | 0.10 |
| MRC2 | 5.31 | 0.02 | 778.16 | 0.44 | 0.22 | 0.89 | 0.02 |
| MRGPRF | 10.73 | 0.00 | 30.66 | 0.26 | 0.03 | 1.26 | 0.12 |
| MS4A3 | 9.18 | 0.00 | 286.91 | 0.51 | 0.22 | 1.12 | 0.10 |
| MST1P2 | 4.69 | 0.03 | 36.74 | 0.42 | 0.20 | 0.86 | 0.02 |
| MST1 | 4.04 | 0.04 | 129.23 | 0.73 | 0.31 | 1.74 | 0.48 |
| MUC16 | 4.47 | 0.03 | 55.25 | 0.36 | 0.05 | 2.01 | 0.26 |
| MXRA7 | 8.16 | 0.00 | 597.81 | 0.48 | 0.19 | 1.20 | 0.11 |
| NAALADL1 | 6.92 | 0.01 | 216.81 | 0.24 | 0.10 | 0.52 | 0.00 |
| NANOS1 | 10.21 | 0.00 | 168.12 | 0.45 | 0.18 | 1.09 | 0.08 |
| NBPF3 | 6.80 | 0.01 | 218.26 | 0.44 | 0.21 | 0.92 | 0.03 |
| NCRNA00204B | 6.15 | 0.01 | 29.42 | 0.41 | 0.18 | 0.89 | 0.03 |
| NDFIP2 | 4.06 | 0.04 | 84.97 | 0.55 | 0.26 | 1.17 | 0.12 |
| NNMT | 8.07 | 0.00 | 10.37 | 0.51 | 0.25 | 1.04 | 0.06 |
| NOD1 | 8.38 | 0.00 | 844.18 | 0.35 | 0.16 | 0.76 | 0.01 |
| NOMO1 | 7.01 | 0.01 | 3135.42 | 0.50 | 0.20 | 1.24 | 0.13 |
| NOMO2 | 4.70 | 0.03 | 1524.27 | 0.65 | 0.32 | 1.32 | 0.23 |
| NOMO3 | 8.55 | 0.00 | 1489.76 | 0.70 | 0.32 | 1.58 | 0.39 |
| NTNG2 | 10.27 | 0.00 | 1268.11 | 0.27 | 0.10 | 0.71 | 0.01 |
| NUCB2 | 13.23 | 0.00 | 7093.21 | 0.36 | 0.16 | 0.82 | 0.01 |
| NUDT10 | 6.36 | 0.01 | 87.42 | 0.42 | 0.18 | 1.00 | 0.05 |
| OGDHL | 10.75 | 0.00 | 14.85 | 0.39 | 0.14 | 1.08 | 0.07 |
| ORMDL3 | 5.34 | 0.02 | 573.28 | 0.42 | 0.20 | 0.87 | 0.02 |
| OSBPL5 | 12.12 | 0.00 | 2443.75 | 0.24 | 0.08 | 0.65 | 0.01 |
| P2RX6 | 4.52 | 0.03 | 8.57 | 0.38 | 0.18 | 0.80 | 0.01 |
| P2RY8 | 7.19 | 0.01 | 3928.90 | 0.40 | 0.19 | 0.82 | 0.01 |
| P4HB | 6.24 | 0.01 | 14471.56 | 0.47 | 0.18 | 1.20 | 0.11 |
| PCBP3 | 12.34 | 0.00 | 265.41 | 0.33 | 0.11 | 0.90 | 0.03 |
| PCBP4 | 7.09 | 0.01 | 238.36 | 0.21 | 0.06 | 0.65 | 0.01 |
| PDE3B | 18.13 | 0.00 | 2424.25 | 0.34 | 0.16 | 0.71 | 0.00 |
| PDIA3P | 14.17 | 0.00 | 2116.12 | 0.24 | 0.08 | 0.67 | 0.01 |
| PDIA3 | 13.09 | 0.00 | 7840.28 | 0.28 | 0.09 | 0.78 | 0.02 |
| PDIA4 | 7.98 | 0.00 | 3004.33 | 0.53 | 0.22 | 1.28 | 0.15 |
| PDIA5 | 5.52 | 0.02 | 362.87 | 0.54 | 0.24 | 1.24 | 0.14 |
| PDIA6 | 11.24 | 0.00 | 5506.68 | 0.31 | 0.12 | 0.81 | 0.02 |
| PHYHD1 | 4.13 | 0.04 | 42.68 | 0.54 | 0.24 | 1.23 | 0.13 |
| PKP4 | 7.50 | 0.01 | 1885.63 | 0.38 | 0.14 | 1.03 | 0.06 |
| PLA2G4A | 25.85 | 0.00 | 386.15 | 3.88 | 1.84 | 8.46 | 0.00 |
| PLCH1 | 10.13 | 0.00 | 494.98 | 0.26 | 0.05 | 1.00 | 0.07 |
| POM121L1P | 8.40 | 0.00 | 15.29 | 0.57 | 0.23 | 1.45 | 0.24 |
| POU6F1 | 7.62 | 0.01 | 301.38 | 0.22 | 0.05 | 0.79 | 0.03 |
| PRRT4 | 11.54 | 0.00 | 175.36 | 0.44 | 0.21 | 0.90 | 0.02 |
| PRSS12 | 4.17 | 0.04 | 8.45 | 0.43 | 0.20 | 0.87 | 0.02 |
| PURG | 11.76 | 0.00 | 22.23 | 0.24 | 0.09 | 0.59 | 0.00 |
| PXK | 11.08 | 0.00 | 1334.81 | 0.78 | 0.38 | 1.62 | 0.50 |
| RAB5B | 10.65 | 0.00 | 4609.74 | 0.43 | 0.14 | 1.27 | 0.13 |
| RABL5 | 5.22 | 0.02 | 88.09 | 0.65 | 0.32 | 1.32 | 0.23 |
| RCN1 | 7.72 | 0.01 | 1430.99 | 0.49 | 0.23 | 1.06 | 0.07 |
| RETN | 7.43 | 0.01 | 85.46 | 0.44 | 0.21 | 0.93 | 0.03 |
| RFX8 | 10.03 | 0.00 | 605.95 | 0.44 | 0.20 | 0.95 | 0.04 |
| RGS3 | 12.33 | 0.00 | 1684.13 | 0.24 | 0.07 | 0.72 | 0.01 |
| RGS9BP | 13.17 | 0.00 | 39.21 | 0.25 | 0.09 | 0.63 | 0.00 |
| RILPL1 | 10.74 | 0.00 | 139.47 | 0.16 | 0.02 | 0.75 | 0.03 |
| RNF145 | 10.85 | 0.00 | 434.80 | 5.08 | 1.13 | 36.29 | 0.05 |
| RPGR | 11.22 | 0.00 | 580.44 | 0.62 | 0.27 | 1.43 | 0.26 |
| RPN2 | 5.10 | 0.02 | 7241.73 | 0.38 | 0.16 | 0.89 | 0.03 |
| RPUSD3 | 8.86 | 0.00 | 1055.50 | 0.58 | 0.20 | 1.67 | 0.31 |
| RREB1 | 10.50 | 0.00 | 5270.06 | 0.64 | 0.30 | 1.34 | 0.23 |
| RTN4R | 6.28 | 0.01 | 310.59 | 0.32 | 0.15 | 0.67 | 0.00 |
| S100B | 14.15 | 0.00 | 398.76 | 0.28 | 0.09 | 0.76 | 0.02 |
| SAMD11 | 10.97 | 0.00 | 41.60 | 0.30 | 0.14 | 0.64 | 0.00 |
| SCCPDH | 10.76 | 0.00 | 1822.52 | 0.47 | 0.21 | 1.05 | 0.06 |
| SERPING1 | 4.89 | 0.03 | 316.61 | 0.25 | 0.08 | 0.63 | 0.01 |
| SIX3 | 18.57 | 0.00 | 1.12 | 0.40 | 0.18 | 0.88 | 0.02 |
| SLC13A5 | 4.30 | 0.04 | 1.36 | 0.38 | 0.18 | 0.82 | 0.01 |
| SLC17A7 | 6.28 | 0.01 | 5.55 | 0.52 | 0.18 | 1.42 | 0.20 |
| SLC1A4 | 5.83 | 0.02 | 1736.50 | 0.88 | 0.43 | 1.85 | 0.74 |
| SLC22A16 | 16.39 | 0.00 | 537.59 | 0.65 | 0.31 | 1.35 | 0.24 |
| SLC24A3 | 16.52 | 0.00 | 326.93 | 0.28 | 0.13 | 0.63 | 0.00 |
| SLC25A38 | 7.14 | 0.01 | 761.57 | 0.57 | 0.25 | 1.31 | 0.18 |
| SLC26A9 | 7.10 | 0.01 | 136.70 | 0.00 | NA | ####### | 0.99 |
| SLC28A3 | 12.78 | 0.00 | 32.22 | 0.37 | 0.17 | 0.81 | 0.01 |
| SLC30A3 | 5.50 | 0.02 | 1.60 | 0.44 | 0.21 | 0.88 | 0.02 |
| SLC35C2 | 5.23 | 0.02 | 1179.39 | 0.51 | 0.24 | 1.09 | 0.08 |
| SLC39A11 | 8.57 | 0.00 | 679.17 | 0.49 | 0.23 | 1.01 | 0.05 |
| SLPI | 10.63 | 0.00 | 203.22 | 0.34 | 0.11 | 0.99 | 0.05 |
| SPRY2 | 10.19 | 0.00 | 627.59 | 0.42 | 0.20 | 0.86 | 0.02 |
| SSTR2 | 17.06 | 0.00 | 17.74 | 0.29 | 0.12 | 0.70 | 0.01 |
| ST3GAL4 | 4.27 | 0.04 | 1321.15 | 0.74 | 0.34 | 1.66 | 0.46 |
| ST3GAL6 | 12.42 | 0.00 | 314.35 | 0.32 | 0.12 | 0.75 | 0.01 |
| STAB1 | 4.55 | 0.03 | 3930.56 | 2.31 | 1.13 | 4.78 | 0.02 |
| STAR | 14.18 | 0.00 | 233.10 | 0.35 | 0.17 | 0.71 | 0.00 |
| STIM2 | 20.19 | 0.00 | 608.41 | 3.29 | 1.55 | 7.33 | 0.00 |
| STOX2 | 9.52 | 0.00 | 24.13 | 0.29 | 0.13 | 0.64 | 0.00 |
| STXBP1 | 6.34 | 0.01 | 267.28 | 0.41 | 0.17 | 0.96 | 0.04 |
| STYK1 | 6.76 | 0.01 | 114.50 | 0.46 | 0.18 | 1.17 | 0.10 |
| SULT1C2 | 5.46 | 0.02 | 0.93 | 0.55 | 0.27 | 1.09 | 0.09 |
| SUMO1P1 | 4.72 | 0.03 | 4.61 | 0.41 | 0.18 | 0.93 | 0.03 |
| TARM1 | 7.83 | 0.01 | 9.08 | 0.44 | 0.19 | 1.02 | 0.06 |
| TASP1 | 7.15 | 0.01 | 165.64 | 0.61 | 0.28 | 1.30 | 0.21 |
| TBC1D2B | 11.60 | 0.00 | 3446.34 | 0.37 | 0.14 | 0.93 | 0.04 |
| TBX1 | 16.20 | 0.00 | 25.23 | 0.22 | 0.10 | 0.47 | 0.00 |
| TCTN1 | 5.41 | 0.02 | 137.88 | 0.60 | 0.28 | 1.28 | 0.19 |
| TDRD9 | 19.92 | 0.00 | 131.38 | 0.27 | 0.12 | 0.58 | 0.00 |
| TEPP | 8.03 | 0.00 | 4.54 | 0.33 | 0.12 | 0.91 | 0.03 |
| TEX101 | 19.95 | 0.00 | 0.89 | 0.23 | 0.10 | 0.52 | 0.00 |
| TGFA | 10.85 | 0.00 | 105.06 | 0.38 | 0.17 | 0.80 | 0.01 |
| TGIF1 | 18.97 | 0.00 | 687.40 | 0.20 | 0.09 | 0.44 | 0.00 |
| TLE1 | 6.62 | 0.01 | 222.16 | 0.51 | 0.25 | 1.07 | 0.07 |
| TM6SF1 | 7.82 | 0.01 | 127.44 | 0.43 | 0.20 | 0.92 | 0.03 |
| TMEM50B | 9.48 | 0.00 | 1212.54 | 0.45 | 0.18 | 1.10 | 0.08 |
| TMEM87A | 10.84 | 0.00 | 1383.48 | 0.52 | 0.24 | 1.14 | 0.10 |
| TMX4 | 6.21 | 0.01 | 1200.35 | 0.60 | 0.30 | 1.21 | 0.16 |
| TNNI3 | 7.75 | 0.01 | 1.35 | 0.43 | 0.21 | 0.91 | 0.03 |
| TOR1A | 11.38 | 0.00 | 1749.42 | 0.44 | 0.15 | 1.21 | 0.11 |
| TRAF3IP2 | 8.97 | 0.00 | 759.20 | 0.42 | 0.16 | 1.07 | 0.07 |
| TRPM6 | 11.61 | 0.00 | 22.13 | 0.30 | 0.11 | 0.80 | 0.02 |
| TWIST1 | 17.94 | 0.00 | 9.97 | 0.32 | 0.11 | 0.87 | 0.03 |
| UBE2Q2 | 17.41 | 0.00 | 1280.95 | 0.57 | 0.25 | 1.26 | 0.17 |
| UGT3A2 | 12.88 | 0.00 | 490.10 | 0.33 | 0.11 | 0.94 | 0.04 |
| VCL | 9.85 | 0.00 | 2511.14 | 2.94 | 1.26 | 7.00 | 0.01 |
| VSTM1 | 10.59 | 0.00 | 269.03 | 0.39 | 0.19 | 0.83 | 0.01 |
| WIT1 | 6.99 | 0.01 | 204.50 | 0.55 | 0.27 | 1.11 | 0.09 |
| WT1 | 9.28 | 0.00 | 530.02 | 0.28 | 0.11 | 0.69 | 0.01 |
| WWC2 | 12.78 | 0.00 | 321.87 | 0.40 | 0.18 | 0.90 | 0.03 |
| XKR5 | 6.47 | 0.01 | 36.29 | 0.44 | 0.19 | 1.00 | 0.05 |
| ZBTB7C | 6.04 | 0.01 | 10.91 | 0.52 | 0.21 | 1.29 | 0.16 |
| ZDHHC23 | 5.93 | 0.01 | 964.32 | 0.75 | 0.29 | 1.96 | 0.55 |
| ZDHHC9 | 3.91 | 0.05 | 411.21 | 0.67 | 0.30 | 1.44 | 0.31 |
| ZNF498 | 13.05 | 0.00 | 572.87 | 0.57 | 0.27 | 1.17 | 0.13 |
| ZNF697 | 10.85 | 0.00 | 117.63 | 0.35 | 0.17 | 0.73 | 0.01 |
| ZSCAN22 | 5.43 | 0.02 | 301.54 | 0.39 | 0.18 | 0.84 | 0.02 |
| ACIN1 | 6.09 | 0.01 | 7037.16 | 0.26 | 0.11 | 0.57 | 0.00 |
| ADAMTS13 | 4.33 | 0.04 | 95.52 | 0.34 | 0.14 | 0.81 | 0.01 |
| ANKS3 | 5.52 | 0.02 | 413.45 | 0.27 | 0.11 | 0.64 | 0.00 |
| C9orf96 | 5.13 | 0.02 | 27.36 | 0.34 | 0.15 | 0.72 | 0.01 |
| CDC42BPG | 4.06 | 0.04 | 141.88 | 0.32 | 0.14 | 0.73 | 0.01 |
| COQ10A | 10.41 | 0.00 | 202.42 | 0.29 | 0.12 | 0.70 | 0.01 |
| CUL9 | 8.45 | 0.00 | 2192.77 | 0.29 | 0.12 | 0.69 | 0.01 |
| DCLRE1B | 5.06 | 0.02 | 270.21 | 3.23 | 1.44 | 7.37 | 0.00 |
| DIS3L2 | 6.53 | 0.01 | 672.03 | 0.43 | 0.20 | 0.90 | 0.03 |
| FLJ35220 | 16.79 | 0.00 | 142.49 | 0.17 | 0.07 | 0.42 | 0.00 |
| GAPDHS | 4.04 | 0.04 | 3.27 | 1.58 | 0.78 | 3.24 | 0.20 |
| HEMK1 | 6.54 | 0.01 | 972.71 | 0.37 | 0.18 | 0.73 | 0.01 |
| HLA | 11.09 | 0.00 | 12.12 | 0.24 | 0.11 | 0.50 | 0.00 |
| HSFX2 | 10.22 | 0.00 | 17.31 | 0.25 | 0.12 | 0.53 | 0.00 |
| IFT122 | 11.07 | 0.00 | 1032.05 | 0.19 | 0.08 | 0.43 | 0.00 |
| IFT27 | 8.21 | 0.00 | 966.96 | 0.29 | 0.13 | 0.63 | 0.00 |
| IL11RA | 4.99 | 0.03 | 797.91 | 0.22 | 0.07 | 0.66 | 0.01 |
| ITFG2 | 4.73 | 0.03 | 759.20 | 0.36 | 0.17 | 0.73 | 0.01 |
| L3MBTL | 5.25 | 0.02 | 524.87 | 0.40 | 0.19 | 0.81 | 0.01 |
| LMAN2L | 7.98 | 0.00 | 481.89 | 0.31 | 0.14 | 0.69 | 0.00 |
| LMF1 | 7.61 | 0.01 | 363.97 | 0.23 | 0.10 | 0.53 | 0.00 |
| LOC100132247 | 4.40 | 0.04 | 1421.69 | 0.72 | 0.34 | 1.52 | 0.40 |
| LOC283922 | 6.57 | 0.01 | 546.39 | 0.40 | 0.20 | 0.81 | 0.01 |
| LOC339047 | 6.55 | 0.01 | 2696.94 | 0.23 | 0.07 | 0.66 | 0.01 |
| LRP5L | 6.43 | 0.01 | 303.88 | 0.44 | 0.19 | 0.95 | 0.04 |
| LRRC48 | 5.59 | 0.02 | 47.44 | 0.30 | 0.10 | 0.89 | 0.03 |
| MAMDC4 | 7.63 | 0.01 | 350.55 | 0.24 | 0.10 | 0.56 | 0.00 |
| MSH5 | 7.96 | 0.00 | 1397.05 | 0.74 | 0.32 | 1.64 | 0.47 |
| MUM1 | 9.54 | 0.00 | 1074.53 | 0.44 | 0.21 | 0.96 | 0.04 |
| NPHP4 | 6.95 | 0.01 | 765.73 | 0.47 | 0.18 | 1.21 | 0.12 |
| NTN5 | 4.39 | 0.04 | 20.76 | 0.43 | 0.20 | 0.89 | 0.03 |
| OBSL1 | 7.01 | 0.01 | 745.60 | 0.35 | 0.17 | 0.72 | 0.00 |
| PAN2 | 6.31 | 0.01 | 2180.65 | 0.24 | 0.10 | 0.56 | 0.00 |
| PIGO | 5.56 | 0.02 | 1023.11 | 0.49 | 0.24 | 0.99 | 0.05 |
| PILRB | 4.32 | 0.04 | 3093.23 | 0.41 | 0.20 | 0.87 | 0.02 |
| POLG2 | 9.25 | 0.00 | 521.86 | 0.31 | 0.14 | 0.70 | 0.01 |
| POLR2J4 | 7.06 | 0.01 | 767.04 | 0.40 | 0.19 | 0.79 | 0.01 |
| POLR3E | 7.65 | 0.01 | 1087.76 | 0.25 | 0.11 | 0.59 | 0.00 |
| POMT1 | 9.79 | 0.00 | 941.58 | 0.20 | 0.07 | 0.54 | 0.00 |
| POMT2 | 7.27 | 0.01 | 472.69 | 0.37 | 0.16 | 0.88 | 0.02 |
| PVRIG | 5.53 | 0.02 | 170.95 | 0.33 | 0.16 | 0.67 | 0.00 |
| RABL2A | 5.03 | 0.02 | 507.40 | 0.36 | 0.16 | 0.79 | 0.01 |
| SFRS8 | 4.11 | 0.04 | 1878.31 | 3.45 | 0.85 | 23.58 | 0.13 |
| SGK2 | 5.08 | 0.02 | 34.07 | 0.30 | 0.13 | 0.67 | 0.00 |
| SMG6 | 4.71 | 0.03 | 908.46 | 0.44 | 0.17 | 1.03 | 0.07 |
| SPG7 | 5.02 | 0.02 | 1812.18 | 0.27 | 0.12 | 0.60 | 0.00 |
| SPPL2B | 5.70 | 0.02 | 2588.07 | 0.30 | 0.13 | 0.70 | 0.01 |
| SPRN | 5.09 | 0.02 | 176.07 | 0.37 | 0.18 | 0.77 | 0.01 |
| TBC1D3 | 6.38 | 0.01 | 1000.88 | 0.09 | 0.02 | 0.34 | 0.00 |
| USP20 | 4.29 | 0.04 | 778.12 | 2.57 | 1.07 | 6.26 | 0.04 |
| VAMP1 | 8.16 | 0.00 | 1228.80 | 0.37 | 0.18 | 0.77 | 0.01 |
| WDR6 | 4.41 | 0.04 | 5508.91 | 0.35 | 0.15 | 0.81 | 0.01 |
| ZNF446 | 4.37 | 0.04 | 160.57 | 0.43 | 0.21 | 0.89 | 0.02 |
| ZNF497 | 9.33 | 0.00 | 345.56 | 0.43 | 0.16 | 1.13 | 0.09 |
| ZNF517 | 7.31 | 0.01 | 241.33 | 0.33 | 0.15 | 0.70 | 0.00 |
| ZNF839 | 11.32 | 0.00 | 515.91 | 0.21 | 0.08 | 0.52 | 0.00 |

Notes: OR: odd ratio, CI: confidence interval

Supplementary Table3. Association between the clinicopathologic characteristics and patients’ mortality of AML patients in the TCGA dataset

| Variables | Group | Alive | Dead | P value | Statistical method |
| --- | --- | --- | --- | --- | --- |
| Age |  | 48 | 59.04 | 0.00 | Wilcoxon sum rank test |
| PBMBC |  | 37.12 | 39.75 | 0.62 | Wilcoxon sum rank test |
| White blood cell |  | 33.7 | 39 | 0.41 | Wilcoxon sum rank test |
| Gender | Female | 26 | 54 | 0.75 | Fisher’s exact test |
|  | Male | 33 | 60 |  |  |
| Cytogenetic risk | Favorable | 20 | 12 | 0.00 | Fisher’s exact test |
|  | Intermediate | 30 | 72 |  |  |
|  | Poor | 8 | 28 |  |  |
| *IDH1* mutation | Mutant | 7 | 9 | 0.42 | Fisher’s exact test |
|  | Wild-type | 52 | 105 |  |  |
| *IDH2* mutation | Mutant | 5 | 12 | 0.79 | Fisher’s exact test |
|  | Wild-type | 54 | 102 |  |  |
| *DNMT3A* mutation | Mutant | 10 | 33 | 0.1 | Fisher’s exact test |
|  | Wild-type | 49 | 81 |  |  |
| *NPM1* mutation | Mutant | 15 | 33 | 0.72 | Fisher’s exact test |
|  | Wild-type | 44 | 81 |  |  |
| *CEBPA* mutation | Mutant | 4 | 9 | 1 | Fisher’s exact test |
|  | Wild-type | 55 | 105 |  |  |
| *FLT3* mutation | Mutant | 15 | 34 | 0.6 | Fisher’s exact test |
|  | Wild-type | 44 | 80 |  |  |
| Neoadjuvant treatment | Yes | 12 | 33 | 0.27 | Fisher’s exact test |
|  | No | 47 | 81 |  |  |

Supplementary Table4. Association between the clinicopathologic characteristics and patients’ mortality of AML patients in the validation dataset

| Variables | Group | Alive | Dead | P value | Statistical method |
| --- | --- | --- | --- | --- | --- |
| Age |  | 49.23 | 61.84 | 0.00 | Wilcoxon sum rank test |
| PBMBC |  | 60.01 | 58.7 | 0.72 | Wilcoxon sum rank test |
| Gender | Female | 82 | 95 | 0.07 | Fisher’s exact test |
|  | Male | 85 | 143 |  |  |
| Cytogenetic risk | Favorable | 76 | 41 | 0.00 | Fisher’s exact test |
|  | Intermediate | 48 | 94 |  |  |
|  | Poor | 42 | 103 |  |  |
| FLT3-IDT | Negative | 134 | 176 | 0.12 | Fisher’s exact test |
|  | Positive | 32 | 62 |  |  |
| CEBPA mutation | Negative | 67 | 83 | 1 | Fisher’s exact test |
|  | Positive | 12 | 14 |  |  |
| *IDH1* mutation | Negative | 67 | 89 | 0.3 | Fisher’s exact test |
|  | Positive | 14 | 11 |  |  |
| *DNMT3A* mutation | Negative | 41 | 54 | 0.87 | Fisher’s exact test |
|  | Positive | 25 | 31 |  |  |
| *NPM1* mutation | Negative | 123 | 179 | 0.73 | Fisher’s exact test |
|  | Positive | 43 | 57 |  |  |
| Chemotherapy | Yes | 164 | 212 | 0.01 | Fisher’s exact test |
|  | No | 1 | 14 |  |  |
| Bone marrow transplant | Yes | 70 | 42 | 0.00 | Fisher’s exact test |
|  | No | 95 | 184 |  |  |
| Targeted therapy | Yes | 16 | 55 | 0.00 | Fisher’s exact test |

Supplementary Table5. Kaplan-Meier survival analysis and multivariate analysis between gene expression and OS of AML patients

in the validation dataset

|  | Kaplan-Meier survival analysis | | | Multivariate analysis | | | |
| --- | --- | --- | --- | --- | --- | --- | --- |
| Gene | chisq | P value | Cutoff | OR | 2.50%CI | 97.50%CI | P value |
| ACCS | 6.83 | 0.01 | 6.23 | 0.49 | 0.26 | 0.93 | 0.03 |
| AGRN | 14.98 | 0.00 | 2.60 | 0.49 | 0.30 | 0.79 | 0.00 |
| ASNS | 8.24 | 0.00 | 3.67 | 0.59 | 0.36 | 0.96 | 0.03 |
| C14orf64 | 9.30 | 0.00 | 1.75 | 0.37 | 0.21 | 0.62 | 0.00 |
| C9orf43 | 8.24 | 0.00 | 4.58 | 0.56 | 0.32 | 0.99 | 0.04 |
| CALR | 5.18 | 0.02 | 10.80 | 0.34 | 0.21 | 0.55 | 0.00 |
| CDH26 | 5.83 | 0.02 | 2.01 | 0.44 | 0.26 | 0.73 | 0.00 |
| CPA6 | 5.98 | 0.01 | 0.39 | 0.53 | 0.33 | 0.84 | 0.01 |
| CYP2E1 | 6.89 | 0.01 | 1.21 | 0.39 | 0.21 | 0.69 | 0.00 |
| DHRS7 | 6.56 | 0.01 | 6.86 | 0.46 | 0.29 | 0.74 | 0.00 |
| DIS3L2 | 4.21 | 0.04 | 3.63 | 0.62 | 0.39 | 0.97 | 0.04 |
| FAM45A | 5.37 | 0.02 | 4.04 | 0.51 | 0.32 | 0.81 | 0.01 |
| FNDC3B | 3.99 | 0.04 | 5.67 | 0.29 | 0.17 | 0.47 | 0.00 |
| GOLGA3 | 7.04 | 0.01 | 5.57 | 0.36 | 0.22 | 0.57 | 0.00 |
| IGDCC4 | 9.24 | 0.00 | 3.10 | 0.55 | 0.34 | 0.87 | 0.01 |
| ITGB6 | 7.74 | 0.01 | 3.72 | 0.44 | 0.28 | 0.69 | 0.00 |
| KRT17 | 10.23 | 0.00 | 2.40 | 0.34 | 0.18 | 0.64 | 0.00 |
| MAMDC4 | 7.33 | 0.01 | 3.31 | 0.49 | 0.31 | 0.77 | 0.00 |
| MBTPS1 | 6.66 | 0.01 | 6.46 | 0.40 | 0.23 | 0.69 | 0.00 |
| MICALL2 | 26.51 | 0.00 | 4.55 | 0.38 | 0.23 | 0.61 | 0.00 |
| NTNG2 | 9.88 | 0.00 | 4.14 | 0.59 | 0.37 | 0.95 | 0.03 |
| OSBPL5 | 5.45 | 0.02 | 5.60 | 0.37 | 0.23 | 0.59 | 0.00 |
| PIGO | 6.62 | 0.01 | 3.94 | 0.60 | 0.37 | 0.95 | 0.03 |
| PILRB | 22.02 | 0.00 | 1.28 | 0.27 | 0.12 | 0.56 | 0.00 |
| PLA2G4A | 10.40 | 0.00 | 4.18 | 2.85 | 1.78 | 4.64 | 0.00 |
| POU6F1 | 8.54 | 0.00 | 2.23 | 0.64 | 0.40 | 0.99 | 0.04 |
| RABL2A | 8.36 | 0.00 | 3.53 | 0.52 | 0.32 | 0.82 | 0.01 |
| RILPL1 | 5.66 | 0.02 | 1.39 | 0.63 | 0.40 | 1.00 | 0.04 |
| RTN4R | 7.95 | 0.00 | 2.76 | 0.60 | 0.38 | 0.96 | 0.03 |
| S100B | 5.66 | 0.02 | 2.79 | 0.61 | 0.38 | 0.96 | 0.03 |
| SSTR2 | 14.21 | 0.00 | 3.81 | 0.50 | 0.26 | 0.91 | 0.03 |
| STAR | 8.62 | 0.00 | 3.88 | 0.46 | 0.29 | 0.73 | 0.00 |
| TBC1D2B | 15.48 | 0.00 | 5.83 | 0.51 | 0.31 | 0.82 | 0.01 |
| TDRD9 | 4.68 | 0.03 | 3.08 | 0.47 | 0.29 | 0.76 | 0.00 |
| TGIF1 | 13.06 | 0.00 | 4.07 | 0.33 | 0.19 | 0.58 | 0.00 |
| VCL | 11.09 | 0.00 | 7.78 | 1.99 | 1.19 | 3.38 | 0.01 |
| VSTM1 | 4.07 | 0.04 | 6.02 | 0.32 | 0.20 | 0.52 | 0.00 |
| ZNF446 | 8.87 | 0.00 | 3.13 | 0.48 | 0.26 | 0.87 | 0.02 |
| ZSCAN22 | 5.94 | 0.01 | 1.95 | 0.55 | 0.35 | 0.87 | 0.01 |

Supplementary table6. The comparison of clinical characteristics among the four subgroups of AML patients in the TCGA dataset

| Variables | Group | Cluster1 | Cluster2 | Cluster3 | Cluster4 | P value | Statistical method |
| --- | --- | --- | --- | --- | --- | --- | --- |
| Age |  | 53.62 | 51.64 | 66.7 | 54.75 | 0.03 | ANOVA test |
| PBMBC |  | 39.77 | 36.21 | 35.96 | 37.92 | 0.62 | ANOVA test |
| Gender | Female | 58 | 8 | 9 | 5 | 0.75 | Fisher’s exact test |
|  | Male | 66 | 6 | 14 | 7 |  |  |
| Cytogenetic risk | Favorable | 30 | 1 | 0 | 1 | 0.03 | Fisher’s exact test |
|  | Intermediate | 71 | 9 | 14 | 9 |  |  |
|  | Poor | 22 | 3 | 9 | 2 |  |  |
| *IDH1* mutation | Mutant | 12 | 1 | 2 | 1 | 1 | Fisher’s exact test |
|  | Wild-type | 112 | 13 | 21 | 11 |  |  |
| *IDH2* mutation | Mutant | 11 | 3 | 2 | 1 | 0.45 | Fisher’s exact test |
|  | Wild-type | 113 | 11 | 21 | 11 |  |  |
| *DNMT3A* mutation | Mutant | 27 | 4 | 5 | 7 | 0.06 | Fisher’s exact test |
|  | Wild-type | 97 | 10 | 18 | 5 |  |  |
| *NPM1* mutation | Mutant | 36 | 3 | 2 | 7 | 0.02 | Fisher’s exact test |
|  | Wild-type | 88 | 11 | 21 | 5 |  |  |
| *CEBPA* mutation | Mutant | 10 | 0 | 2 | 1 | 0.81 | Fisher’s exact test |
|  | Wild-type | 114 | 14 | 21 | 11 |  |  |
| *FLT3* mutation | Mutant | 39 | 5 | 2 | 3 | 0.12 | Fisher’s exact test |
|  | Wild-type | 85 | 9 | 21 | 9 |  |  |
| Neoadjuvant treatment | Yes | 33 | 4 | 3 | 5 | 0.29 | Fisher’s exact test |
|  | No | 91 | 10 | 20 | 7 |  |  |

Note: ANOVA: analysis of variance

Supplementary table7. The comparison of clinical characteristics among the four subgroups of AML patients in the validation dataset

| Variables | Group | Cluster1 | Cluster2 | Cluster3 | Cluster4 | P value | Statistical method |
| --- | --- | --- | --- | --- | --- | --- | --- |
| Age |  | 54.13 | 56.1 | 59.58 | 53.83 | 0.58 | ANOVA test |
| PBMBC |  | 60.33 | 81.92 | 41.72 | 65.43 | 0.01 | ANOVA test |
| Gender | Female | 33 | 54 | 62 | 28 | 0.13 | Fisher’s exact test |
|  | Male | 39 | 49 | 91 | 49 |  |  |
| Cytogenetic risk | Favorable | 18 | 25 | 61 | 41 | 0.00 | Fisher’s exact test |
|  | Intermediate | 29 | 33 | 37 | 18 |  |  |
|  | Poor | 24 | 45 | 55 | 18 |  |  |
| *IDH1* mutation | Mutant | 4 | 11 | 8 | 2 | 0.13 | Fisher’s exact test |
|  | Wild-type | 36 | 33 | 62 | 25 |  |  |
| *DNMT3A* mutation | Mutant | 6 | 18 | 20 | 12 | 0.1 | Fisher’s exact test |
|  | Wild-type | 24 | 19 | 30 | 22 |  |  |
| *NPM1* mutation | Mutant | 11 | 52 | 19 | 18 | 0.00 | Fisher’s exact test |
|  | Wild-type | 60 | 50 | 134 | 58 |  |  |
| *CEBPA* mutation | Mutant | 8 | 4 | 5 | 9 | 0.2 | Fisher’s exact test |
|  | Wild-type | 35 | 41 | 50 | 33 |  |  |
| FLT3-IDT | Mutant | 9 | 44 | 19 | 22 | 0.00 | Fisher’s exact test |
|  | Wild-type | 62 | 59 | 134 | 55 |  |  |
| Chemotherapy | Yes | 68 | 95 | 141 | 72 | 0.72 | Fisher’s exact test |
|  | No | 1 | 5 | 6 | 3 |  |  |
| Bone marrow transplant | Yes | 15 | 39 | 41 | 17 | 0.05 | Fisher’s exact test |
|  | No | 54 | 61 | 106 | 58 |  |  |
| Targeted therapy | Yes | 9 | 20 | 22 | 20 | 0.12 | Fisher’s exact test |
|  | No | 60 | 80 | 125 | 55 |  |  |

Supplementary table8. the associations between FLT3 mutation, NPM1 mutation and overall survival in the TCGA cohort

|  |  | Patients with RNA-seq (n=173) | All patients (n=200) |  |  |
| --- | --- | --- | --- | --- | --- |
| Variables | Group | Alive/Dead | Alive/Dead | P value | Statistical method |
| *FLT3* mutation | Negative | 44/80 | 17/38 | >0.05 for all cases | Fisher’s exact test or Chi-squared test |
|  | Positive | 15/34 | 47/85 |  |  |
| *NPM1* mutation | Negative | 44/81 | 49/86 | >0.05 for all cases | Fisher’s exact test or Chi-squared test |
|  | Positive | 15/33 | 15/37 |  |  |

Supplementary Table9. the associations between FLT3-IDT, NPM1 mutation and overall survival in the validation cohort

|  |  | Patients with RNA-seq (n=405) | All patients (n=672) |  |  |  |
| --- | --- | --- | --- | --- | --- | --- |
| Variables | Group | Alive/ Dead | P value | Alive/ Dead | P value | Statistical method |
| FLT3-IDT | Negative | 134/176 | >0.05 for all cases | 197/231 | 0.002 | Fisher’s exact test or Chi-squared test |
|  | Positive | 32/62 |  | 42/94 |  |  |
| *NPM1* mutation | Negative | 123/179 | >0.05 for all cases | 177/240 | 1 | Fisher’s exact test or Chi-squared test |
|  | Positive | 43/57 |  | 62/85 |  |  |
